# Automated quantification of skin Gb3 load and white matter lesion assessment in Fabry disease

**DOI:** 10.1186/s13023-026-04490-4

**Published:** 2026-07-07

**Authors:** Catharina Pfister, Magnus Schindehütte, Christoph Erbacher, Thomas Klein, Simone Rost, Aljosha Calvin Lang, Mirko Pham, Peter Nordbeck, Christoph Wanner, Claudia Sommer, Nurcan Üçeyler

**Affiliations:** 1 Department of Neurology, University Hospital Würzburg, Josef-Schneider-Str. 11, 97080 Würzburg, Germany; 2 Department of Neuroradiology, University Hospital Würzburg, Würzburg, Germany; 3 Institute of Clinical Genetics and Genome Medicine, University Hospital Würzburg, Würzburg, Germany; 4 Department of Internal Medicine I, University Hospital Würzburg, Würzburg, Germany; 5 Würzburg Fabry Center for Interdisciplinary Therapy (FAZIT), University Hospital Würzburg, Würzburg, Germany; 6 Department of Clinical Studies and Epidemiology, Comprehensive Heart Failure Center, University Hospital Würzburg, Würzburg, Germany; 7 Department of Anesthesiology, University Hospital Würzburg, Würzburg, Germany

**Keywords:** Fabry disease, Skin punch biopsy, Globotriaosylceramide 3, Fabry-specific therapy, Genetics

## Abstract

**Background:**

Fabry disease (FD) is an X-linked lysosomal storage disorder characterized by cellular accumulation of globotriaosylceramide (Gb3).

**Methods:**

We investigated dermal Gb3 load as a potential surrogate marker for systemic FD manifestations by applying an automated image analysis pipeline to skin punch biopsies from the lower leg of 149 individuals carrying variants in *GLA*, the gene encoding alpha-galactosidase A. Variants were stratified by pathogenicity: Group 1 (pathogenic), Group 2 (non-pathogenic), and Group 3 (variants of unknown significance). Gb3 was visualized via Shiga toxin subunit B and quantified using CellProfiler (v4.1.3). Results were compared to healthy controls and patients with idiopathic small fiber neuropathy.

**Results:**

Dermal Gb3 load was elevated in both men and women with pathogenic *GLA* variants (Group 1, *p* < 0.001), excluding those with the N215S variant. Increased Gb3 deposition was associated with pain in men (*p* < 0.05), but not with overall disease severity, treatment status, or cerebral white matter lesions. Diagnostic sensitivity and specificity to correctly determine FD were 67% and 95% in men, and 78% and 81% in women, respectively.

**Conclusion:**

Our findings demonstrate the feasibility of automated dermal Gb3 quantification and its capacity to detect cutaneous Gb3 accumulation in pathogenic *GLA* variant carriers. However, its diagnostic accuracy is moderate, and it does not reliably reflect systemic involvement in FD.

**Supplementary Information:**

The online version contains supplementary material available at 10.1186/s13023-026-04490-4.

## Background

Fabry disease (FD) is an X-linked lysosomal storage disorder caused by pathogenic variants in the *GLA* gene, which encodes the enzyme α-galactosidase A (α-GAL). These variants result in reduced or absent α-GAL enzymatic activity, leading to the progressive accumulation of sphingolipids, predominantly globotriaosylceramide (Gb3), within various tissues and body fluids [1].

The skin, as an easily accessible organ, demonstrates the accumulation of Gb3 within the lysosomes of several cell types [2–7]. Previous studies have demonstrated that Gb3 accumulation in skin correlates with disease severity in FD, primarily using immunohistochemical detection with monoclonal antibodies [8–10]. While informative, this approach relies on indirect antigen recognition and manual or semi-quantitative assessment of histological sections, which may introduce variability related to antibody performance, staining conditions, and observer-dependent interpretation. These limitations can affect reproducibility and restrict applicability in routine clinical settings, highlighting the need for more robust and standardized methods for Gb3 visualization and quantification.

The central nervous system is also involved in FD, presenting with early-onset cerebral stroke and white matter lesions (WML) [11]. WML are detected using cerebral magnetic resonance imaging (cMRI) and are thought to arise from underlying microangiopathy [12]. Although the clinical implications of WML remain uncertain, an increased WML burden has been associated with greater disease severity [13, 14].

Our objective was to leverage readily obtainable skin punch biopsies for the automated quantification of tissue Gb3 load using specific visualization via Shiga toxin [15], and to evaluate its potential as a diagnostic and prognostic biomarker in FD. Specifically, we aimed to investigate whether tissue Gb3 levels could serve as a surrogate for systemic disease burden, including their potential to reflect cerebral WML load as a hallmark of FD-associated central nervous system involvement.

## Methods and patients

### Patients

We have investigated data and biomaterial of adult individuals carrying pathogenic and non-pathogenic variants, as well as variants of unknown significance in the *GLA* gene. Individuals were prospectively seen for baseline and follow-up visits at our Fabry Center for interdisciplinary Therapy (FAZIT), University Hospital Würzburg 2006–2020, and had agreed to a diagnostic skin punch biopsy. As a disease control group, we included patients with idiopathic small fiber neuropathy (SFN) who were diagnosed following current criteria [16] 2014–2020 at the Department of Neurology, University Hospital Würzburg. Additionally, we recruited a healthy adult control group in 2020 at the Department of Neurology, University Hospital Würzburg who had a normal skin innervation, i.e. an intraepidermal nerve fiber density (IENFD) of ≥ 6 fibers/mm on lower leg skin punch biopsies. Study participants gave written informed consent before enrollment.

### Genetic classification

Genetic reports were provided individually when registering for the first interview at FAZIT. *GLA* sequence variants were categorized following the five pathogenicity classes of sequence variants as defined by the American College of Medical Genetics and Genomics (ACMG) [17], namely benign (class 1), likely benign (class 2), variant of unknown significance (class 3), variant of unknown significance with probable pathogenicity (class 3+) [18], likely pathogenic (class 4), and pathogenic (class 5). This was determined using Alamut Visual 2.11 (Interactive Biosoftware, Rouen, France), which integrates data from population and mutation databases, prediction tools, and literature.

### Clinical and laboratory phenotyping

A comprehensive medical history was obtained from all patients and all patients underwent neurological examination. Patients filled in validated pain questionnaires, namely the Neuropathic Pain Symptom Inventory and the Fabry Pain Questionnaire [19, 20]. Enzymatic α-GAL activity was measured in leukocytes, and plasma lyso-Gb3 levels had been quantified. Additional assessments included nerve conduction studies of the sural nerve following a standard procedure to exclude large nerve fiber impairment and quantitative sensory testing (QST) at the back of the foot for sensory profiling [21].

Renal function was assessed using the estimated glomerular filtration rate (eGFR), and albuminuria via the albumin-to-creatinine ratio in morning spot urine (UACR), calculated in accordance with Kidney Disease: Improving Global Outcomes (KDIGO) guidelines [22]. Kidney disease was defined as UACR ≥ 30 mg/g or an eGFR less than 60 ml/min/1.73m^2^.

Cardiac function was evaluated using electrocardiography, echocardiography, and cardiac MRI. FD cardiomyopathy was diagnosed based on imaging criteria indicating an advanced disease stage, with a septal wall thickness ≥ 12 mm or a left ventricular mass index ≥ 95 g/m². Additionally, cardiac MRI was used to assess gadolinium distribution, with findings of intramural, transmural, or disseminated enhancement affecting > 2 myocardial segments also meeting the criteria for FD cardiomyopathy diagnosis.

For the categorization of FD symptom severity, we applied a scoring system as described previously [23]: involvement of kidneys, heart, brain, and the presence of FD-associated pain were scored with 1 point each and a total score of 0 = none, 1 = mild, 2 = moderate, and ≥ 3 severe was calculated.

### Skin punch biopsy

Diagnostic 5-mm skin punch biopsies were obtained following a standardized protocol from the lower leg of FD patients (apparatus by Stiefel GmbH, Offenbach, Germany) at baseline and ≥ 1 follow-up visit [24]. IENFD was determined on 40-µm skin cryosections immunoreacted with antibodies against protein gene product 9.5 (PGP9.5) and following standard counting rules [25, 26].

20-µm skin samples were stained for Gb3 visualization using Shiga toxin subunit B (STx) [15]. STx 1:5.000 was coupled with the fluorophore 555 (StxB::555, Sigma-Aldrich, Munich, Germany) and DAPI was used for nuclear staining. Three skin sections per subject were investigated using fluorescence microscopy (Zeiss Imager M.2 with Colibri 7 LED light source, Zeiss, Oberkochen, Germany) and Zen Blue/Black Edition software (Zeiss, Oberkochen, Germany). We used a wave length of 555 nm for STx and 385 nm for DAPI. Microscopy conditions were kept identical for data comparability and suppl. Fig. 1 illustrates the strategy of individual sample assessment.

For the automated analysis of dermal Gb3 load, we first used ImageJ software version v1.52i (NIH, Bethesda, MD, USA) to differentiate STx and DAPI stains, and applied CellProfiler (Broad Institute, Cambridge, MA, USA) for automated Gb3 quantification (see pipeline in Supplemental Methods). Dermal Gb3 load was assessed within three regions of interest (ROI) as marked by the blue rectangles (500 × 625 μm) in suppl. Fig. 1. The automatically determined area covered with Gb3 signal was averaged from these three ROI and the assessment was performed on three 20-µm sections per subject. Blue rectangles were placed randomly by the investigator to cover maximum dermal area.

### Cerebral MRI (cMRI)

Since brain biopsy is not feasible and MRI is often contraindicated in FD patients with cardiac device implants, we examined whether dermal Gb3 deposits in skin punch biopsies could act as a surrogate for cerebral pathology, given the shared ectodermal origin of skin and brain. Patients underwent cerebral MRI (cMRI) between 2009 and 2020 at the Institute of Neuroradiology, University Hospital Würzburg. Imaging was performed on a 3.0 Tesla MAGNETOM Prisma Fit (Siemens, Erlangen, Germany) during initial evaluations or follow-up of FD patients. All cMRIs included a T2-weighted FLAIR (Fluid Attenuated Inversion Recovery) sequence for clear delineation of WML. From 2013, an additional T1-weighted MP-RAGE (Magnetization Prepared Rapid Acquisition with Gradient Echoes) sequence was implemented to allow differentiation between gray and white matter. Digital DICOM datasets were stored in a centralized online archive. WML load was assessed using the semiquantitative Fazekas score, categorizing lesions on a scale from 0 (none or punctate, age-appropriate) to 3 (confluent lesions) [27]. Manual 3D voxel-based segmentation of FLAIR sequences provided WML volumes, expressed in voxels. For datasets with MP-RAGE sequences (available since 2010), white matter volumes were quantified using automated segmentation via FreeSurfer software (Martinos Center for Biomedical Imaging, Charlestown, MA, USA). The WML-to-white matter ratio was calculated as a percentage to standardize lesion burden across patients.

### Statistical analysis

Statistical analysis was done using IBM SPSS Statistics 27 Software (IBM, Ehningen, Germany). Data was not normally distributed such that we used the non-parametric Mann-Whitney-U test for groupwise comparison. For correlation analysis, we calculated the Spearman correlation coefficient. For long-term follow-up data, we applied linear mixed models. Sensitivity/specificity analysis was done by receiver operating curves. Data were visualized using GraphPad Prism 8 (San Diego, CA, USA). P-values < 0.05 were considered statistically significant.

## Results

### Characterization of study cohort

**Fig. 1:**
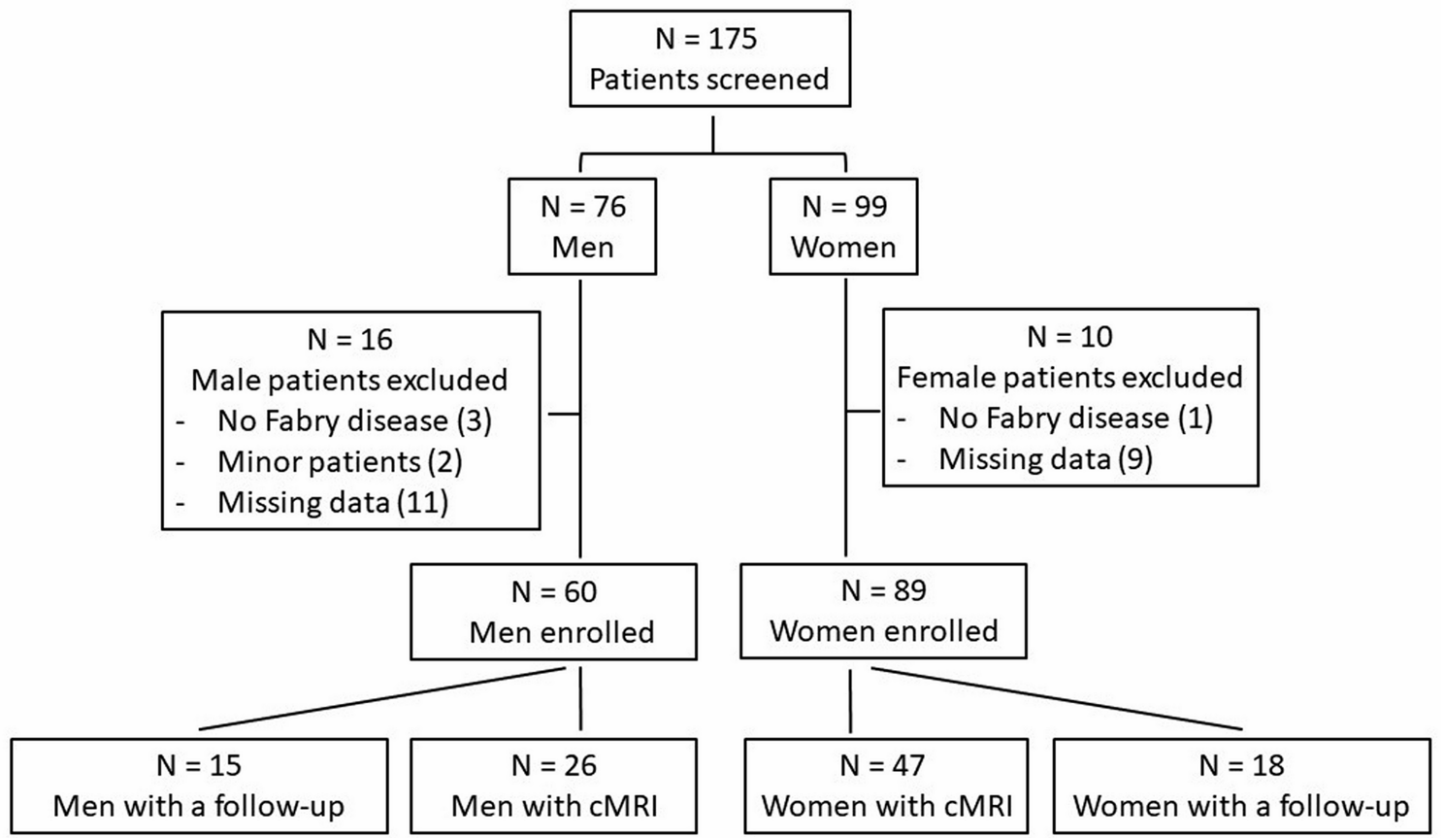
Flow-chart for patient enrolment. The graph shows the original cohort of screened, excluded, and enrolled patients with Fabry disease. Additionally, the number of patients with a follow-up visit and with a cerebral magnetic resonance imaging assessment

We screened data and biomaterial of prospectively recruited 175 adult FD patients and enrolled 149 individuals (60 men; median age 41 years, range 18–71 years; 89 women; median age 44 years, range 18–75 years) as our study cohort (Fig. 1). Data was compared with that of our disease control cohort consisting of 19 patients with idiopathic SFN (8 men; median age 50 years, range 30–64 years; 11 women; median age 51 years, range 31–61 years), and 32 healthy subjects (11 men, median age 32 years, range 24–53 years; 21 women; median age 33 years, range 22–64 years). Detailed clinical and laboratory characteristics are provided in Table 1.

**Table 1:** Clinical characterization of the study population

	Group 1 M	Group 1 F	Group 2 M	Group 2 F	Group 3 M	Group 3 F	Co M	Co F	SFN M	SFN F
N	45	55	5	19	10	15	11	21	8	11
Median age (range) [years]	40 (18–71)	45 (18–71)	49 (34–64)	43 (18–75)	41 (23–64)	43 (26–73)	32 (24–53)	33 (22–64)	50 (30–64)	51 (31–61)
Number of subjects with - Neuropathic pain - Albuminuria - Cardiomyopathy - Cerebral stroke / TIA	29/45 19/45 13/45 2/45	20/55 14/55 22/55 8/55	1/5 1/5 0/5 2/5	7/19 6/19 2/19 5/19	5/10 7/10 9/10 1/10	5/15 6/15 3/15 1/15	NA	NA	8/8	11/11
Median GLA activity (range) [nmol/min/mg] Reference 0,4 − 1 nmol/min/mg	0.03 (0.01–0.42)	0.25 (0,02-6.5)	0.32 (0.11–0.6)	0.44 (0.3–0.51)	0.05 (0.02–0.5)	0.3 (0.13–0.43)	NA	NA	NA	NA
Median lyso-Gb3 (range) [ng/ml] Reference < 0.9 ng/ml	15.9 (0.8–188)	7.9 (0.8–188)	0.6 (0.5–1.57)	0.75 (0.5–2.5)	7.7 (0.5–209)	13 (0.7–17.9)	NA	NA	NA	NA
Number of individuals on ERT	18/45	6/55	0/5	4/19	3/10	7/15	NA	NA	NA	NA
Median time since ERT (range) [years]	3.54	3.81	NA	0.6	4.18	5.58	NA	NA	NA	NA
Number of individuals on chaperon	0/45	0/55	0/5	2/19	0/10	0/15	NA	NA	NA	NA
Median time since chaperon (range) [years]	NA	NA	NA	0.3	NA	4.7	NA	NA	NA	NA
Symptom severity score (N) - No - Mild - Moderate - Severe	3/45 21/45 10/45 11/45	18/55 21/55 7/55 9/55	2/5 2/5 1/5 0/5	6/19 7/19 5/19 1/19	0/10 2/10 6/10 2/10	5/15 8/15 2/15 0/15	NA	NA	NA	NA
Median distal IENFD (range) [fibers/mm]*	3.4	5.7	4.2	6.6	3.2	7.6	7.8	9.9	4.4	4.8

*Laboratory normative values: Lower leg: 9+/-3 fibers/mm

Abbreviations: Co: healthy control, ERT: enzyme replacement therapy, F: female, Gb3: Globotriaosyceramide, GLA: alpha-galactosidase A, IENFD: intraepidermal nerve fiber density, M: male, NA: not available, SFN: small fiber neuropathy, TIA: transient ischemic attack. Group 1: pathogenic *GLA* variants, Group 2: non-pathogenic *GLA* variants, Group 3: variants of unknown significance

### Genetic composition of study group

We stratified our study cohort into “Group 1” (pathogenicity classes 4 and 5 [17], *n* = 45 men and *n* = 55 women), “Group 2” (pathogenicity classes 1 and 2 [17], *n* = 5 men and *n* = 19 women), and “Group 3” (pathogenicity classes 3 and 3+ [17], i.e. variants of unknown significance, VUS; *n* = 10 men and *n* = 15 women). Table 2 presents the genetic distribution of the study population. Individual genotype is provided in suppl. Table 1.

**Table 2:** Genetic classification of study participants

	Men	Women
**Pathogenicity class**^15^ **and group allocation**		
1 = benign (G2)	1/60 (2%)	5/89 (6%)
2 = likely benign (G2)	4/60 (7%)	14/89 (16%)
3 = variant of unknown significance (G3)	5/60 (8%)	10/89 (11%)
3 + = variant of unknown significance likely pathogenic (G3)	5/60 (8%)	5/89 (6%)
4 = likely pathogenic (G1)	10/60 (17%)	13/89 (14%)
5 = pathogenic (G1)	35/60 (58%)	42/89 (47%)
**Variant**		
Missense	37/60 (61%)	56/89 (63%)
Nonsense	9/60 (15%)	9/89 (10%)
Intronic	1/60 (2%)	6/89 (7%)
Frameshift	6/60 (10%)	12/89 (13%)
Deletion	6/60 (10%)	5/89 (6%)
Splice mutation	1/60 (2%)	0/89 (0%)
Upstream substitution	0/60 (0%)	1/89 (1%)

Abbreviations: *GLA*: alpha-galactosidase A gene

G1: pathogenic *GLA* variants, G2: non-pathogenic *GLA* variants, G3: variants of unknown significance

### **Skin Gb3 load is elevated in men carrying pathogenic *GLA *variants**

**Fig. 2:**
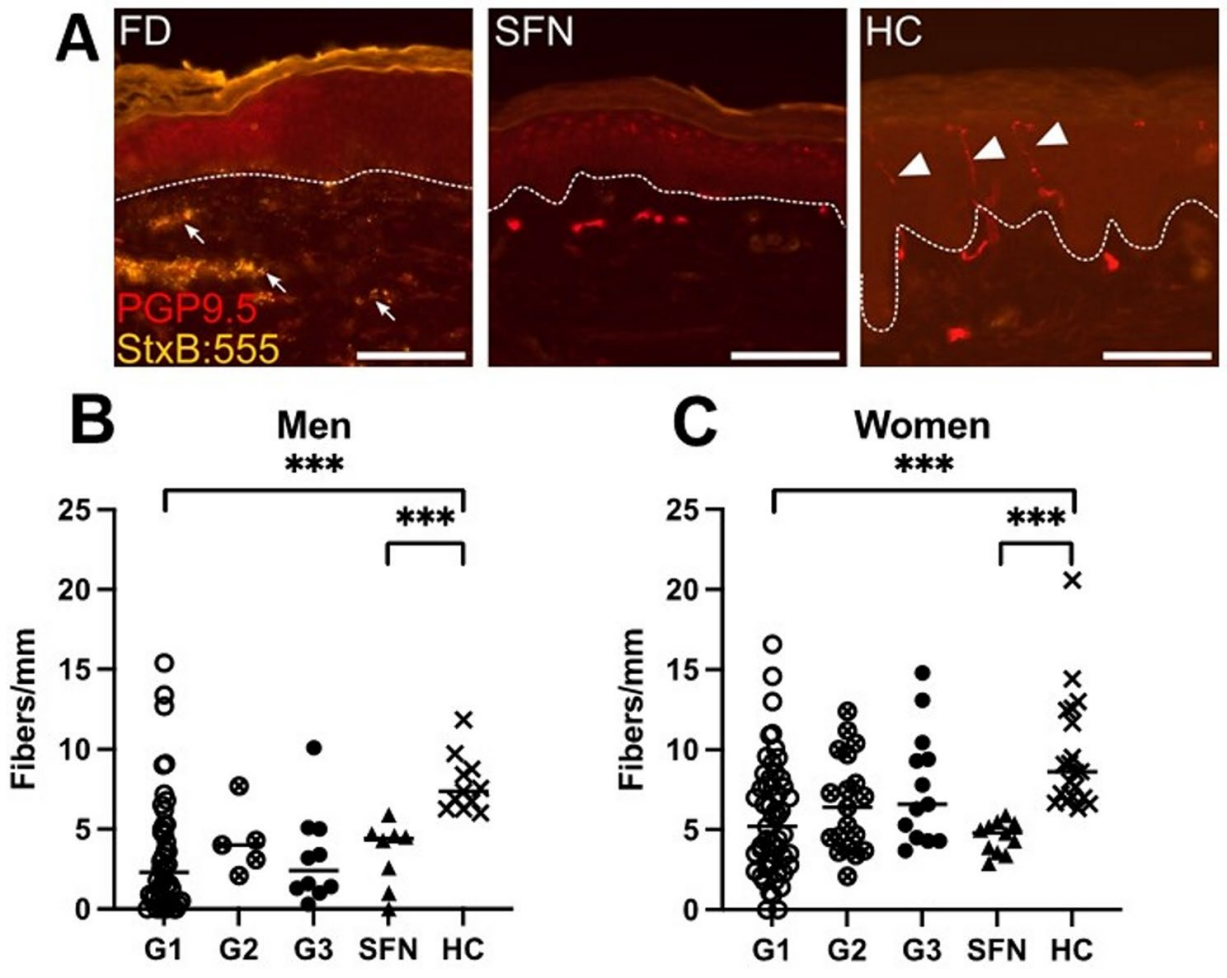
Skin punch biopsy assessment. (**A**) Representative photomicrographs from lateral lower leg biopsies of patients with FD, SFN, and HC. Section  (20 μm) were stained for Gb3 (STxB:555), nerve fibers (PGP 9.5), and nuclei (DAPI). Dermal Gb3 was increased in FD (left; arrows), but absent in SFN (middle), and HC (right). IENFD was normal in HC (arrowheads), but reduced in FD and SFN vs. HC (*p* < 0.001). The epidermis–dermis border is indicated (dotted line). Scale bar = 100 μm. (**B**) and (**C**) Scatter plots of IENFD in FD subgroups (pathogenic [G1], non-pathogenic [G2], VUS [G3]) in men (**B**) and women (**C**), compared to SFN and HC. FD patients with pathogenic variants and SFN patients showed lower IENFD than HC in both sexes. Abbreviations: FD: Fabry disease, SFN: small fiber neuropathy, HC: healthy controls, IENFD: intraepidermal nerve fiber density, Gb3: globotriaosylceramide, VUS: variants of unknown significance. ***p < 0.001

In skin samples, we first investigated distal IENFD and found reduced innervation mainly in men within Group 1 compared to women (*p* < 0.001) and in men and women in Group 1 compared with healthy controls (*p* < 0.001). There were no differences in skin innervation comparing Group 1 with Group 2, Group 3 or disease controls (Table 1; Fig. 2). Skin innervation was normal in women in Groups 2 and 3 (Table 1; Fig. 2).

**Fig. 3:**
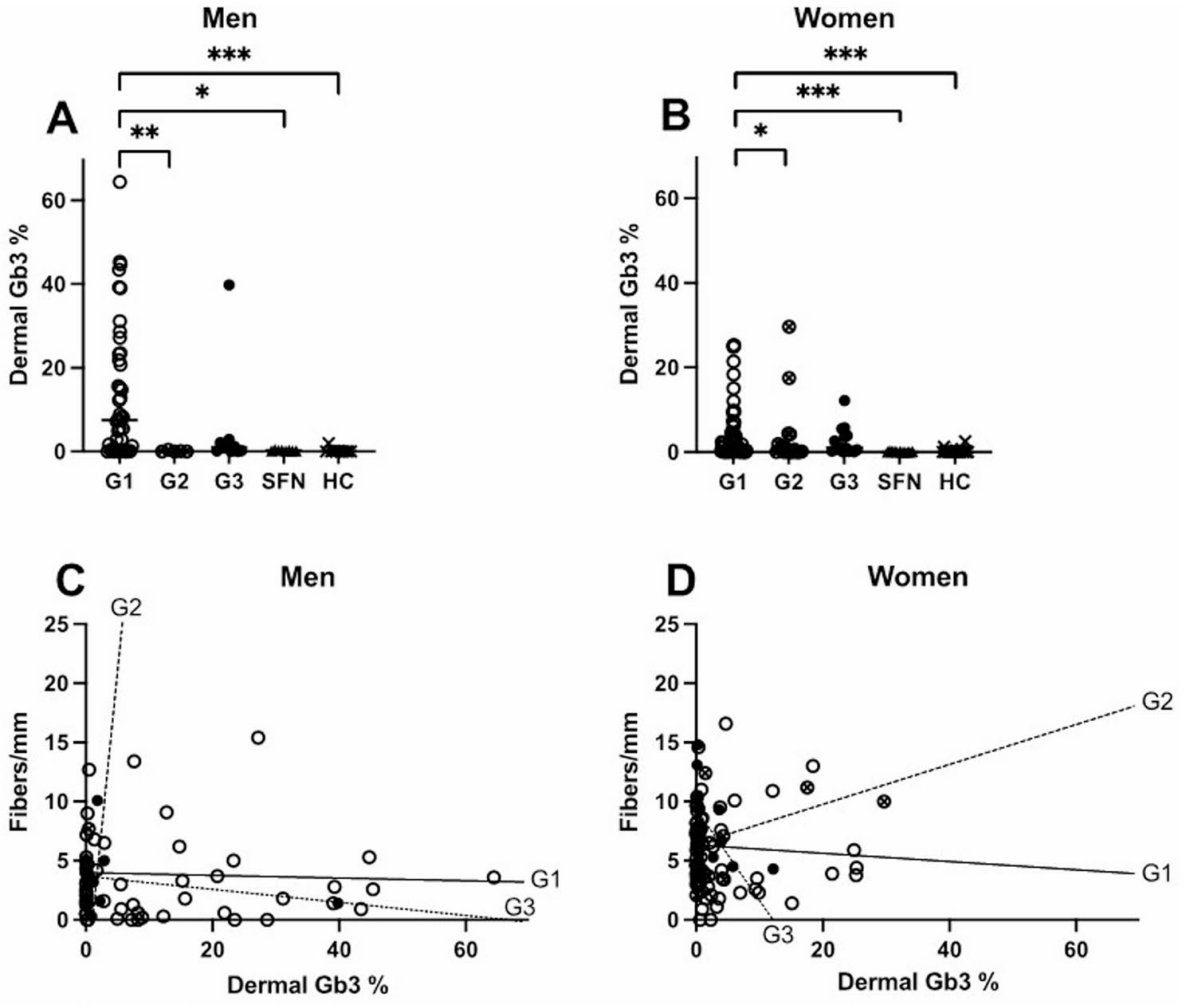
Skin Gb3 and innervation. **A **and **B**) Scatter plots showing dermal Gb3 deposits in FD patients (G1-G3), SFN patients, and HC in men (**A**) and women (**B**). Men and women with a pathogenic variant (G1) had a higher dermal Gb3 load than patients with a non-pathogenic variant (G2; *p* < 0.01 in men, *p* < 0.05 in women), SFN patients (*p* < 0.05 in men, *p* < 0.001 in women), and HC (*p* < 0.001). There was no difference between patients with a pathogenic variant (G1) and patients with a VUS (G3) in men and women. **C** and **D**) Correlation curves between dermal Gb3 and IENFD in men (**C**) and women (**D**) with FD (G1-G3). There was no correlation between dermal Gb3 and IENFD in men and women of all subgroups (G1-G3). Number of subjects per box from left to right: **A**: N = 45, N = 5, N = 10, N = 8, N = 11; **B**: N = 55, N = 20, N = 17, N = 11, N = 21. Number of subjects in groups: Men: G1 = 45, G2 = 5, G3 = 10; women: G1 = 55, G2 = 20; G3 = 17. Abbreviations: FD: Fabry disease, G1: group 1 (pathogenic variants), G2: group 2 (non-pathogenic variants), G3: group 3 (VUS); Gb3: globotriaosylceramide, HC: healthy controls, IENFD: intraepidermal nerve fiber density, SFN: small fiber neuropathy, VUS: variants of unknown significance. *p < 0.05; **p < 0.01; ***p < 0.001

We then analyzed dermal Gb3 load using our automated algorithm and first ensured technical comparability of skin punch biopsy sections. For this, we quantified nuclei of dermal fibroblasts in samples from *n* = 5 randomly selected men and women, each from Group 1, as well as *n* = 3 men and women from both control groups. No intergroup differences were detected, and no correlation was found between fibroblast nuclei count and Gb3 load (*p* > 0.05; suppl. Fig. 2). When investigating skin sections of our study cohort, we found a higher percentage of Gb3 in the dermis of men and women in Group 1 compared to individuals in Group 2 (men: *p* < 0.01; women: *p* < 0.05), to disease controls (men: *p* < 0.05; women: *p* < 0.001), and healthy controls (*p* < 0.001 each). Also, in Group 1, dermal Gb3 load was higher in men than in women (*p* < 0.05; Fig. 3). There was no difference in Gb3 load of men and women in Group 1 compared to Group 3. No correlation was found between Gb3 load and IENFD in all study groups (Fig. 3).

### **Skin Gb3 load is not elevated in men carrying the pathogenic variant N215S**

**Fig. 4:**
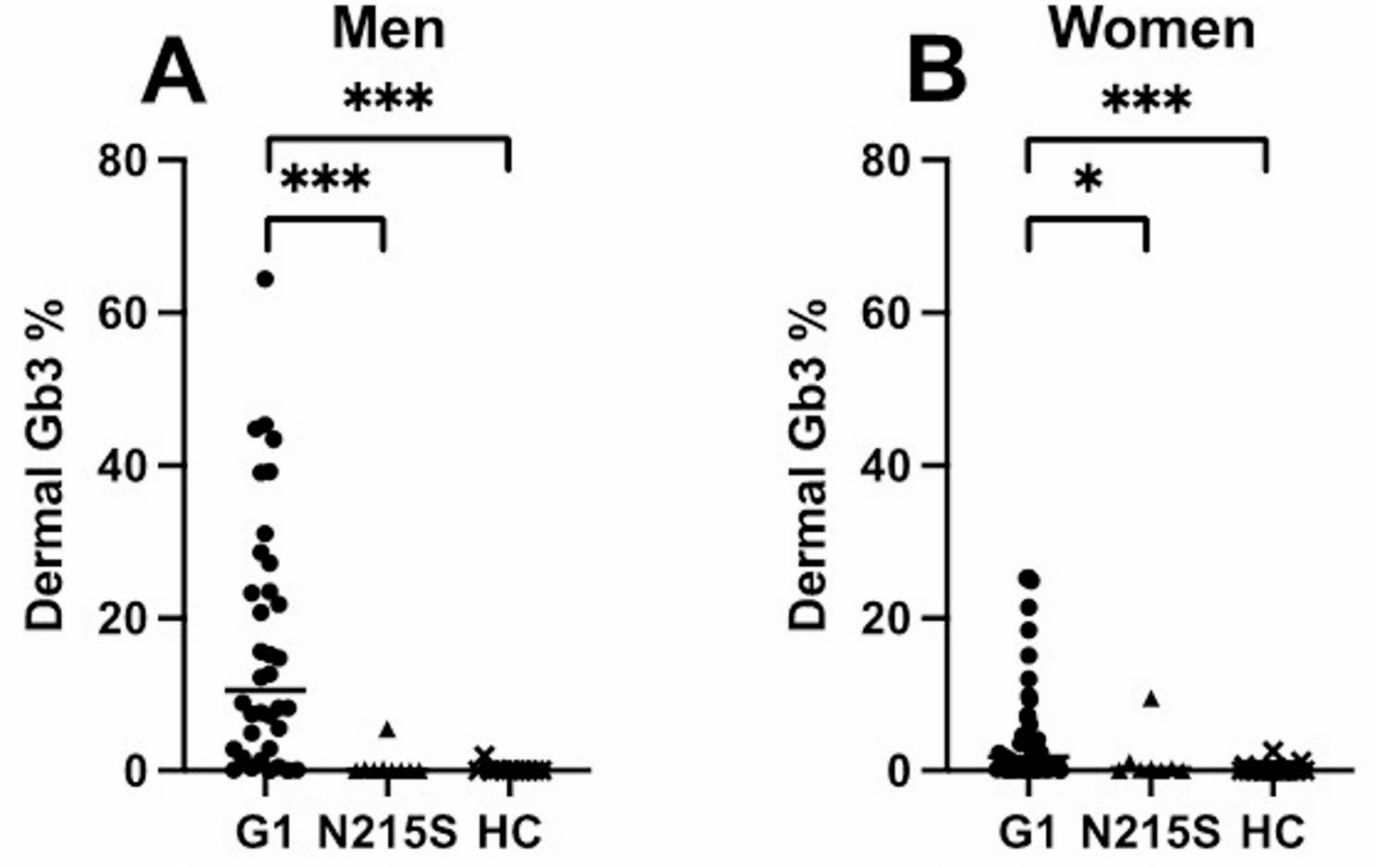
Dermal Gb3 in patients with N215S. Scatter plots showing dermal Gb3 in FD patients with a pathogenic variant (G1), FD patients with an N215S variant, and HC. Men and women with a pathogenic variant had a higher amount of dermal Gb3 than an N215S variant (*p* < 0.001 in men; *p* < 0.05 in women) or HC (*p* < 0.001). Number of subjects per box from left to right: **A**: *N* = 45, *N* = 9, *N* = 11; **B**: *N* = 55, *N* = 8, *N* = 21. Abbreviations FD: Fabry disease, G1: group 1 (pathogenic variants); Gb3: globotriaosylceramide, HC: healthy controls. *p < 0.05; ***p < 0.001

*GLA* variants are mostly “private”, i.e. every FD family has its individual variant, which is reflected by the > 1000 *GLA* variants known to date. Within our study group, the N215S variant was most frequent. Hence, we further studied this specific missense variant, which is typically associated with a cardiac FD phenotype [28]. N215S is a class 5 pathogenic variant with “other” location in the *GLA* gene and clinically “late onset” phenotype. In our cohort, 9/60 (15%) men and 8/89 (9%) women carried the N215S variant. The median disease severity score was 1 for men and for women (range 0–3). Interestingly, despite of its pathogenic character, N215S did not lead to increased dermal Gb3 deposits in men and women compared to other genotypes in Group 1 and to healthy controls (men: *p* < 0.001; women: *p* < 0.05; Fig. 4).

### **Pain is linked to increased dermal Gb3 in men with a pathogenic*GLA*variant**

**Fig. 5:**
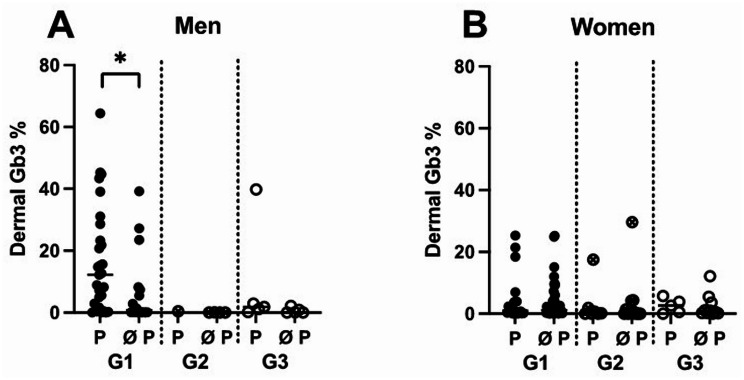
Dermal Gb3 and pain. Scatter plots showing dermal Gb3 in FD patients with pain and without pain with a pathogenic variant (G1), non-pathogenic variant (G2), and VUS (G3). There was a higher dermal Gb3 load in male FD patients with a pathogenic variant and pain than without pain (*p* < 0.05). There were no differences in the other groups in male FD patients and all groups in women FD patients. Number of subjects per box from left to right: **A**: *N* = 29, *N* = 16, *N* = 1, *N* = 4, *N* = 5, *N* = 5; **B**: *N* = 20, *N* = 35, *N* = 7, *N* = 12, *N* = 5, *N* = 10. Abbreviations: FD: Fabry disease, G1: group 1 (pathogenic variants), G2: group 2 (non-pathogenic variants), G3: group 3 (VUS), Gb3: globotriaosylceramide, HC: healthy controls, P: pain, VUS: variants of unknown significance. Ø P: no pain. *p < 0.05

In Group 1, men with currently present FD-associated pain exhibited a higher dermal Gb3 load compared to those without pain (*p* < 0.05; Fig. 5). There was no such difference in men in Group 2 and 3. In women, there was no difference in Gb3 load in either group (Fig. 5).

### Disease severity is not associated with dermal Gb3 load

**Fig. 6:**
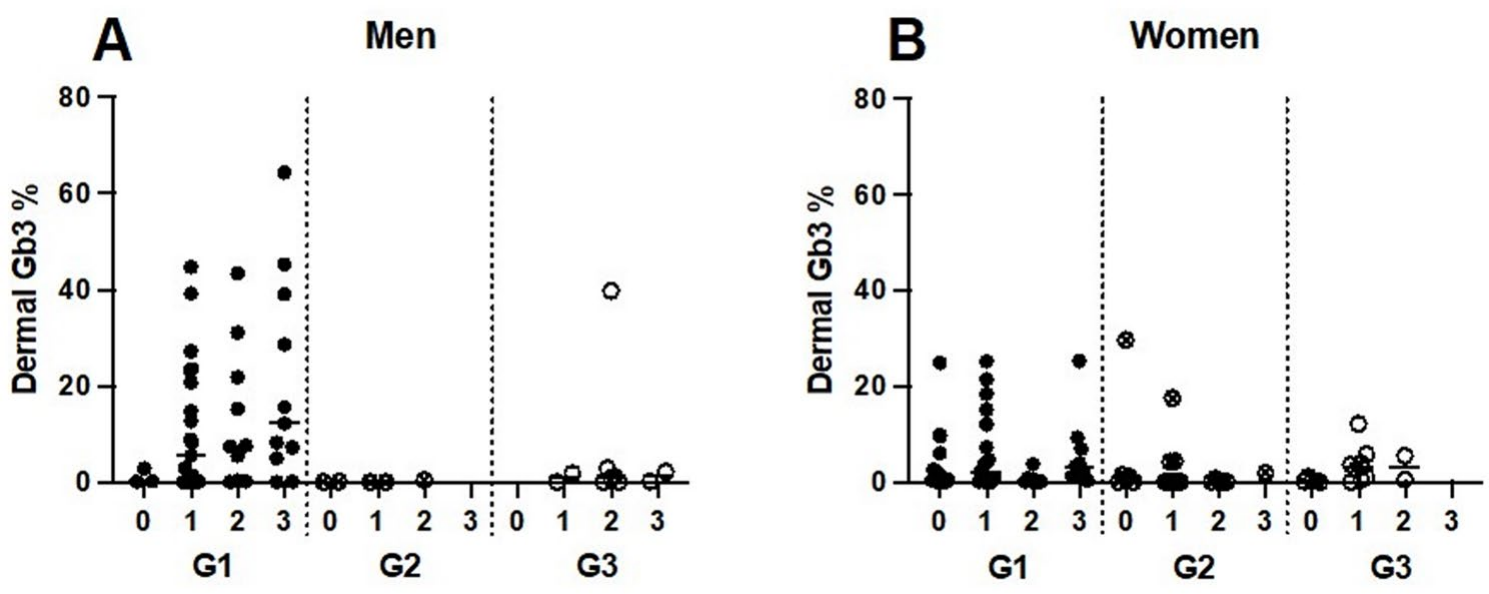
Dermal Gb3 load and disease severity. Scatter plots showing the dermal Gb3 in men and women with FD with a pathogenic variant (G1), non-pathogenic variant (G2), and VUS (G3) and different severity scores from 0 to 3. There were no differences in Gb3 load between the different severity scores in men and women in all subgroups. Number of subjects per box from left to right: **A**: *N* = 3, *N* = 21, *N* = 10, *N* = 11, *N* = 2, *N* = 2; *N* = 1, *N* = 0, *N* = 0, *N* = 2, *N* = 6, *N* = 2 **B**: *N* = 18, *N* = 21, *N* = 7, *N* = 9, *N* = 6, *N* = 7, *N* = 5, *N* = 1, *N* = 5, *N* = 8, *N* = 2, *N* = 1. Abbreviations: FD: Fabry disease, G1: group 1 (pathogenic variants); G2: group 2 (non-pathogenic variants); G3: group 3 (VUS); Gb3: globotriaosylceramide; HC: healthy controls, VUS: variants of unknown significance. 0: no symptoms, 1: mild symptoms, 2: moderate symptoms, 3 severe symptoms

In Group 1, men and women with severe symptoms showed a similar dermal Gb3 load compared to those with no or mild symptoms (Table 3; Fig. 6). In Groups 2 and 3, no correlations were found between clinical symptom severity and dermal Gb3 load irrespective of sex.

**Table 3:** Symptom severity in study cohort

	Men	Women
**Group 1**		
None	3/45 (7%)	18/55 (33%)
Mild	21/45 (47%)	21/55 (38%)
Moderate	10/45 (22%)	7/55 (13%)
Severe	11/45 (24%)	9/55 (16%)
**Group 2**		
None	2/5 (40%)	6/19 (32%)
Mild	2/5 (40%)	7/19 (37%)
Moderate	1/5 (20%)	5/19 (26%)
Severe	0/5 (0%)	1/19 (5%)
**Group 3**		
None	0/10 (0%)	5/15 (33%)
Mild	2/10 (20%)	8/15 (54%)
Moderate	6/10 (60%)	2/15 (13%)
Severe	2/10 (20%)	0/15 (0%)

### Treatment does not alter dermal Gb3 load

**Fig. 7:**
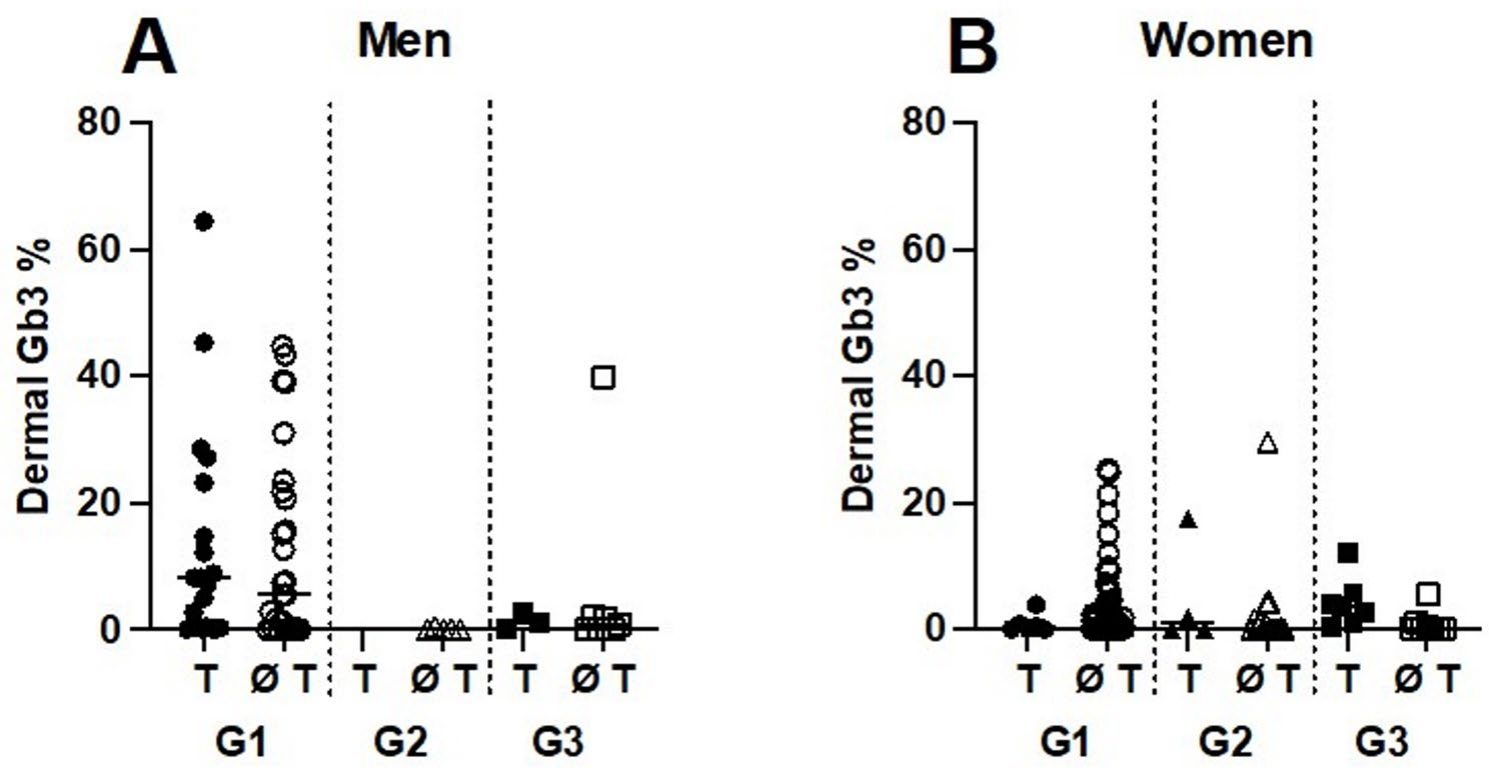
Dermal Gb3 and therapy. Scatter plots showing the dermal Gb3 load in FD patients with and without therapy with a pathogenic variant (G1), non-pathogenic variant (G2), and VUS (G3). There were no differences in Gb3 load between all subgroups in men and women with or without therapy. Number of subjects per box from left to right: **A**: *N* = 18, *N* = 27, *N* = 0, *N* = 5, *N* = 3, *N* = 7; **B**: *N* = 6, *N* = 49, *N* = 4, *N* = 15, *N* = 7, *N* = 8. Abbreviations: FD: Fabry disease, G1: group 1 (pathogenic variants), G2: group 2 (non-pathogenic variants), G3: group 3 (VUS), Gb3: globotriaosylceramide, HC: healthy controls, T: therapy, VUS: variants of unknown significance, Ø T: no therapy

Table 1 provides details of the FD-specific treatment (i.e. enzyme replacement therapy, chaperone) used by the study participants, including treatment depending on symptom severity. FD-specific treatment did not alter dermal Gb3 load over time in patients in Groups 1–3 and between men and women (Table 1; Fig. 7).

### Skin Gb3 load does not decrease during long-term follow-up

In the entire study group, 15/60 (25%) men and 18/89 (20%) women underwent more than one biopsy during follow-up visits. Among these, 2/15 (13%) men and 11/18 (58%) women had not received FD-specific therapy at any biopsy time point. Consistent therapy across biopsies was observed in 6/15 (40%) men and 1/18 (5%) woman, while 7/19 (39%) men and 6/18 (32%) women underwent therapy switch, discontinuation, or initiation between biopsies. Supplementary Table 1 details the frequency of follow-up biopsies per year after baseline. Results were analyzed separately for patients without FD-specific therapy, those with constant therapy, and those with therapy switch. Across all groups, skin Gb3 levels showed increases, decreases, or stability over time, regardless of FD-specific therapy status or sex (suppl. Table 2).

### Diagnostic utility of skin Gb3 load is limited

**Fig. 8:**
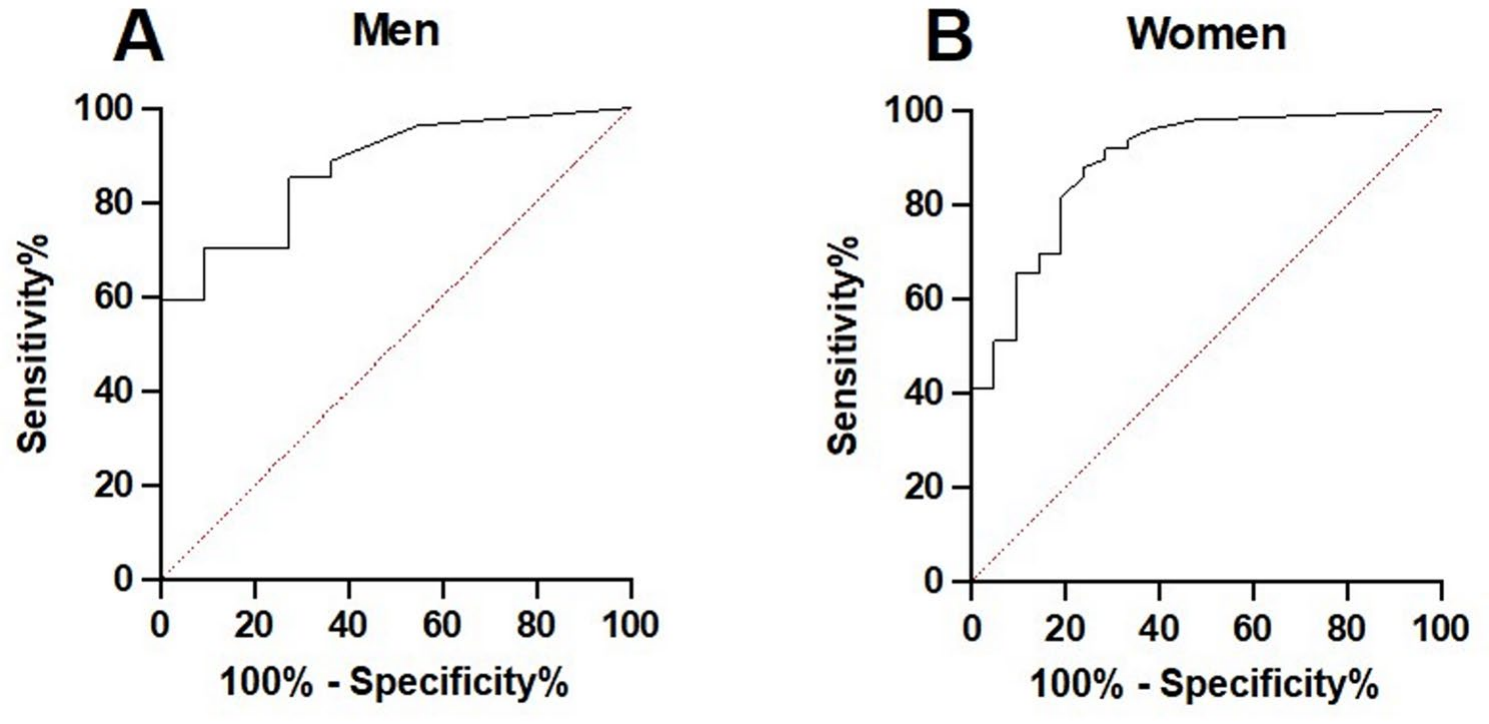
ROC curve of dermal Gb3. The ROC curves show the sensitivity and specificity of men and women with a pathogenic variant (G1) without FD-specific therapy. For men, the AUC was 0.8737, and for women, it was 0.8897. Men had a lower sensitivity (67%) and a higher specificity (95%), while women had a sensitivity of 78% and a specificity of 81%. Number of subjects from left to right: **A**: *N* = 27; **B**: *N* = 49. Abbreviations: AUC: area under the curve, FD: Fabry disease, G1: group 1 (pathogenic variants), Gb3: globotriaosylceramide, ROC: receiver operating characteristic

To determine the effect size of the diagnostic utility of skin Gb3 load in FD, receiver operating characteristic (ROC) analysis was performed, including patients with a pathogenic variant who were not receiving FD-specific therapy. At the time of biopsy, 27/60 (45%) men and 49/89 (55%) women (Group 1 without ERT) met these criteria. The area under the curve (AUC) was 0.8737 for men and 0.8897 for women (Fig. 8). The closest-to-top-left point indicated a sensitivity of 67% and a specificity of 95% for men, and a sensitivity of 78% and a specificity of 81% for women (Group 1 without ERT).

### Skin Gb3 load does not correlate with cMRI WML load in FD

**Fig. 9:**
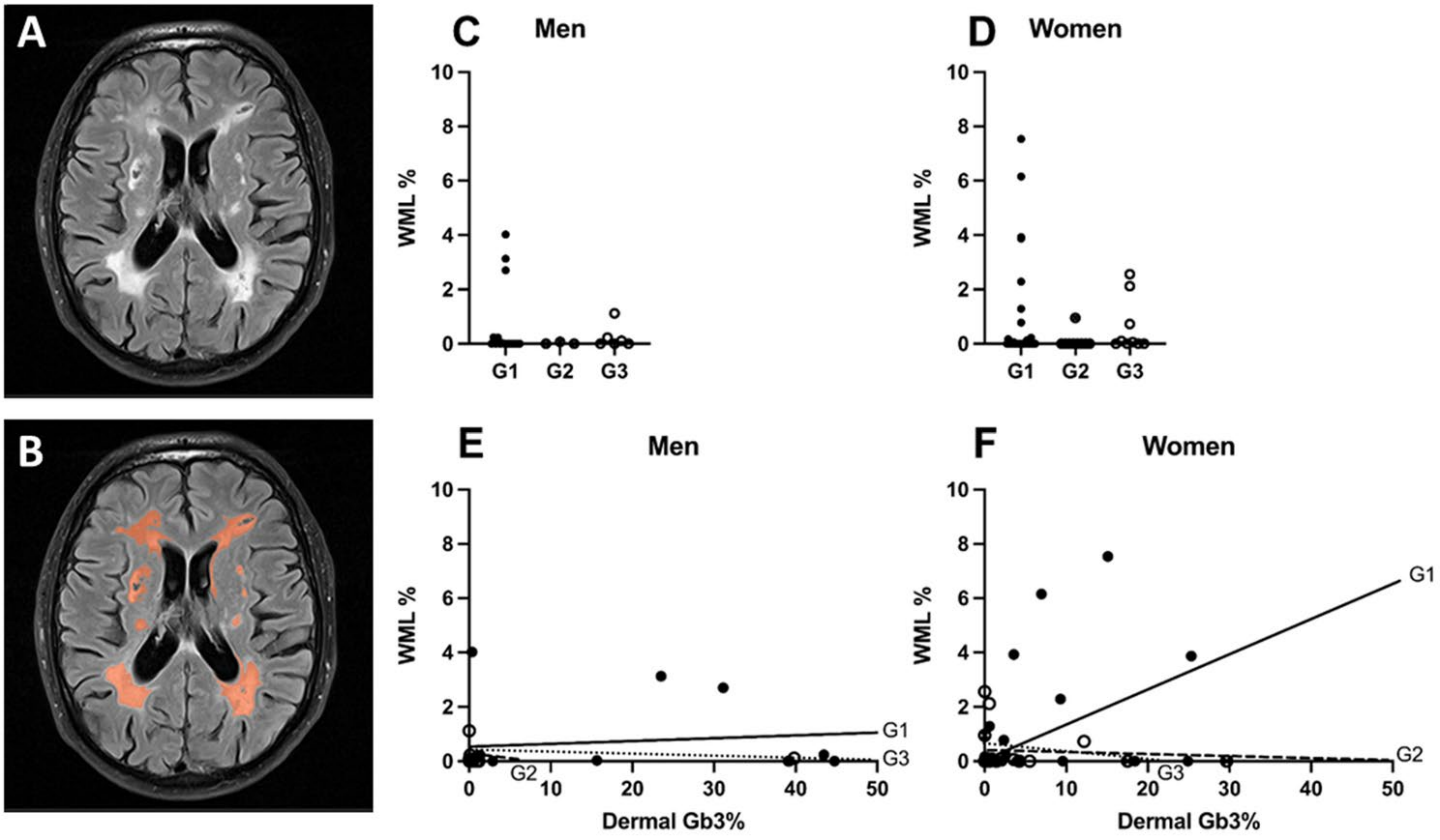
Dermal Gb3 and WML. **A **and **B**) The images show a layer of a cMRI before (**A**) and after (**B**) highlighting the WML in the marking program. **C **and **D**) Scatter plots showing the percentage of WML in FD patients with a pathogenic variant (G1), non-pathogenic variant (G2), and VUS (G3). There were no differences in WML between the subgroups in men and women. **E** and **F**) Correlation curves show the correlation between the dermal Gb3 load and cerebral WML in FD patients with a pathogenic variant (G1), non-pathogenic variant (G2), and VUS (G3). There were no correlations between dermal Gb3 and cerebral WML in all subgroups in men and women. Number of subjects from **C** to **F** and left to right: **C**: N=17, N=3, N=6; **D**: N= 29, N= 9, N= 9; **E**: N=17, N=3, N=6; **F**: N= 29, N= 9, N= 9. Abbreviations: cMRI: cerebral magnetic resonance imaging, FD: Fabry disease, G1: group 1 (pathogenic variants), G2: group 2 (non-pathogenic variants), G3: group 3 (VUS), Gb3: globotriaosylceramide, VUS: variants of unknown significance, WML: white matter lesions

In the entire study group, 26/60 (43%) men and 47/89 (53%) women had also undergone diagnostic cMRI. Table 4 provides stratified cMRI data for Groups 1–3. 16/26 (62%) men and 26/47 (55%) women did not show any WML, while in 4/26 (15%) men and 12/47 (26%) women, WML of moderate to high load was found when applying the Fazekas score (Fig. 9). When we quantified the volume of WML on cMRI scans of 26/60 (43%) men and 47/89 (53%) women, we found the majority of WML (71/73, 97%) ≤ 5% in all subgroups and without intergroup difference (Table 4). We then correlated dermal Gb3 load data with cerebral WML volumes. No correlations were found in men and women within Group 1 between dermal Gb3 deposits and cerebral WML. Also, no correlation was found between dermal Gb3 deposits and WML in individuals within Groups 2 and 3 regardless of sex (Fig. 9).

**Table 4:** Qualitative (Fazekas score) and quantitative (volumetry) analysis of white matter lesions on cMRI scans of study cohort

Group allocation	G1		G2		G3	
Sex	M	F	M	F	M	F
**Fazekas score**						
**0**	11/45	16/55	2/5	6/19	3/10	4/15
1	3/45	7/55	1/5	0/19	2/10	2/15
2	2/45	5/55	0/5	2/19	1/10	3/15
3	1/45	1/55	0/5	1/19	0/10	0/15
No cMRI*	28/45	26/55	2/5	10/19	4/10	6/15
**Volumetry**						
0–1%	14/45	23/55	3/5	9/19	5/10	7/15
1–5%	3/45	4/55	0/5	0/19	1/10	2/15
5–10%	0/45	2/55	0/5	0/19	0/10	0/15
> 10%	0/45	0/45	0/5	0/19	0/10	0/15
No cMRI*	28/45	26/55	2/5	10/19	4/10	6/15

Abbreviations: cMRI: cranial magnetic resonance imaging, F: female, M: male

G1: pathogenic *GLA* variants, G2: non-pathogenic *GLA* variants, G3: variants of unknown significance

*Data from these patients were not included in the denominators for the qualitative and quantitative analysis of white matter lesions

## Discussion

We have established an automated protocol for the quantification of dermal Gb3 accumulation in diagnostic skin punch biopsies and evaluated the potential of cutaneous Gb3 deposits as a surrogate marker in FD. By stratifying our cohort based on the pathogenicity of the respective *GLA* variants, we further underscore the relevance of genetic classification for disease characterization in FD.

Several studies have demonstrated reduced epidermal innervation in both men and women with FD [29, 30], reflecting the underlying small fiber degeneration characteristic of the condition. In our study, which focused on distal skin punch biopsies, this reduction in IENFD was observed exclusively in individuals carrying a pathogenic *GLA* variant. These findings further support the pathophysiological relevance of *GLA* variant stratification in FD [31].

In previous studies, histological assessment of tissue Gb3 accumulation predominantly relied on immunohistochemical detection using anti-Gb3 antibodies [8–10]. Building on our earlier work [8, 15], we employed the B subunit of Shiga toxin as a more specific approach for Gb3 visualization in human skin tissue. To minimize investigator bias, we further implemented an automated image analysis pipeline for the quantification of Gb3 deposits. While Shiga toxin specificity is not entirely exclusive to Gb3, our findings confirm its suitability for the reliable detection of Gb3 in skin biopsy samples [15]. The application of our automated microscopy algorithm proved to be a rapid and robust method for high-throughput analysis and may serve as a valuable tool for the histopathological evaluation of Gb3 in diagnostic skin biopsies. In contrast to antibody-based immunostaining, our approach leverages the natural high-affinity interaction between Shiga toxin and Gb3, enabling direct and highly specific visualization of Gb3 deposits. This represents a conceptual advantage, as it reduces dependence on antibody-related variables such as epitope accessibility and staining efficiency. Importantly, the implementation of automated image analysis further addresses a major limitation of previous methodologies by minimizing observer-dependent bias and enabling objective, reproducible quantification. While earlier studies have demonstrated the clinical utility of Gb3 immunostaining [32], our method provides a more standardized and scalable framework that may be well suited for longitudinal assessment and broader clinical application.

Although *GLA* variants are predominantly private, with most families harboring unique mutations, certain variants have been associated with specific organ manifestations. The *GLA* variant p.Asn215Ser (N215S) is one of the most frequently observed pathogenic variant with more than 20 submissions in ClinVar (https://www.ncbi.nlm.nih.gov/clinvar/variation/10730/) and is predominantly associated with a cardiac phenotype [28]. Although classified as pathogenic, N215S was not associated with dermal Gb3 accumulations in our cohort. While previous reports have suggested possible extracardiac involvement in N215S carriers [33–35], our findings highlight the necessity of evaluating Gb3 deposition in clinically affected tissues to more accurately delineate variant-specific disease manifestations.

We observed a positive correlation between dermal Gb3 load and the presence of pain in men carrying pathogenic *GLA* variants, with higher Gb3 levels detected in the skin of affected individuals reporting pain compared to those without. While our study design does not allow for direct pathophysiological conclusions, we hypothesize that Gb3 accumulation in dermal fibroblasts may contribute to peripheral nociceptor activation [36], potentially via stimulation of the neuro-cutaneous unit [37].

To explore whether dermal Gb3 load reflects clinical phenotype and disease severity, we applied a simplified clinical scoring system encompassing cardiac, renal, and neurological involvement [38]. While Gb3 deposits in the skin were predominantly observed in individuals carrying pathogenic *GLA* variants, we found no relevant differences in Gb3 load when comparing patients with absent, mild, or severe symptomatology. It is important to note that potential associations may have remained undetected due to the cross-sectional nature of our study design. Another potential reason for the lack of correlation between dermal Gb3 accumulation and the semi-quantitative symptom score may lie in limitations of the scoring system itself. The score applied in this study comprises a limited number of clinical parameters and has not been formally validated, which may restrict its ability to adequately capture disease severity in FD, which is of marked phenotypic heterogeneity. In contrast, established severity indices such as the Disease Severity Scoring System (DS3) or the Mainz Severity Score Index (MSSI) incorporate a broader range of clinical domains and organ system involvement. Consequently, the absence of correlation observed in our analysis may reflect insufficient sensitivity of the applied symptom score rather than a true lack of association between dermal Gb3 burden and overall disease severity in FD. This limitation should be considered when interpreting the findings.

We further investigated whether dermal Gb3 load changes in response to FD-specific treatment. Although skin is easily accessible and skin punch biopsy is a minimally invasive procedure, limited data exist on the correlation between dermal Gb3 deposits and overall disease severity in FD. Several previous studies have explored the effect of ERT on tissue Gb3 deposits. For instance, one early study demonstrated Gb3 clearance from skin on the back 20 weeks after ERT initiation in a placebo-controlled trial [39]. Subsequent studies showed partial clearance of dermal Gb3 in both short-term and long-term settings [40, 41] or did not find changes [8]. Gb3 deposits were also semi-quantitatively assessed via electron microscopy in treatment-naïve pediatric male FD patients [42].

In our study, we did not observe a relevant difference in dermal Gb3 load between patients receiving FD-specific treatment and those who do not. Additionally, we found no consistent changes in Gb3 accumulation over time in serial skin biopsies. While a short-term, direct effect of FD-specific therapy on tissue Gb3 burden has been well established [36], our findings are not unexpected given the temporal and potentially tissue-specific nature of treatment efficacy. Moreover, the heterogeneity of Gb3 accumulation observed in our follow-up data may reflect varying durations of treatment prior to biopsy. Contributing factors to these findings likely include the small number of patients within each subgroup and the broad range of treatment durations, from newly initiated ERT to nearly a decade of ongoing therapy. These limitations precluded biologically meaningful statistical comparisons. Therefore, systematic studies in larger, well-characterized cohorts stratified by treatment duration will be essential to clarify the effects of ERT on dermal Gb3 deposition.

Finally, we evaluated the diagnostic utility of dermal Gb3 load in FD and observed only low to moderate sensitivity and specificity. Nonetheless, assessment of Gb3 in skin punch biopsies may hold value for intra-individual longitudinal monitoring, particularly when considering factors such as the interval since the last treatment.

FD patients, particularly those with a high disease burden, may exhibit cerebral involvement in terms of WML. Given the inaccessibility of brain tissue for biopsy and the contraindications to MRI in many FD patients due to implanted cardiac device implants, we investigated whether dermal Gb3 accumulation in skin punch biopsies might serve as a surrogate marker for cerebral pathology, considering that both skin and brain derive from the ectoderm. First, we confirmed our previous observation of only mild to moderate cerebral involvement in our cohort [43], as reflected by both Fazekas scores and quantitative volumetric analysis of WML burden. Although the pathophysiology of WML remains incompletely elucidated [12], our data provide no evidence supporting a direct correlation between dermal Gb3 load and cerebral WML load.

## Conclusion

Our study is limited by its cross-sectional design, which restricts causal inferences and may have obscured subtle associations between dermal Gb3 load and clinical parameters. Additionally, small subgroup sizes and heterogeneous treatment durations limited the statistical power for detecting treatment effects on Gb3 deposition. Still, our work highlights the potential of automated dermal Gb3 quantification as a complementary tool for individualized monitoring in FD, paving the way for more precise tissue-based biomarkers in this complex disorder. 

## Supplementary Information

Below is the link to the electronic supplementary material.

Supplementary Material 1

Supplementary Material 2

Supplementary Material 3

Supplementary Material 4

Supplementary Material 5

## Data Availability

The datasets used and/or analysed during the current study are available from the corresponding author on reasonable request.

## Abbreviations

α-GAL: α-galactosidase a enzyme
FD: Fabry disease
Gb3: Globotriaosylceramide
GLA: Alpha-galactosidase a gene
