# Supplementary figures and images for: Automated quantification of skin Gb3 load and white matter lesion assessment in Fabry disease
Source: Orphanet J Rare Dis. 2026 Jul 7;21:245. doi: 10.1186/s13023-026-04490-4 (PMC13355356; doi:10.1186/s13023-026-04490-4)

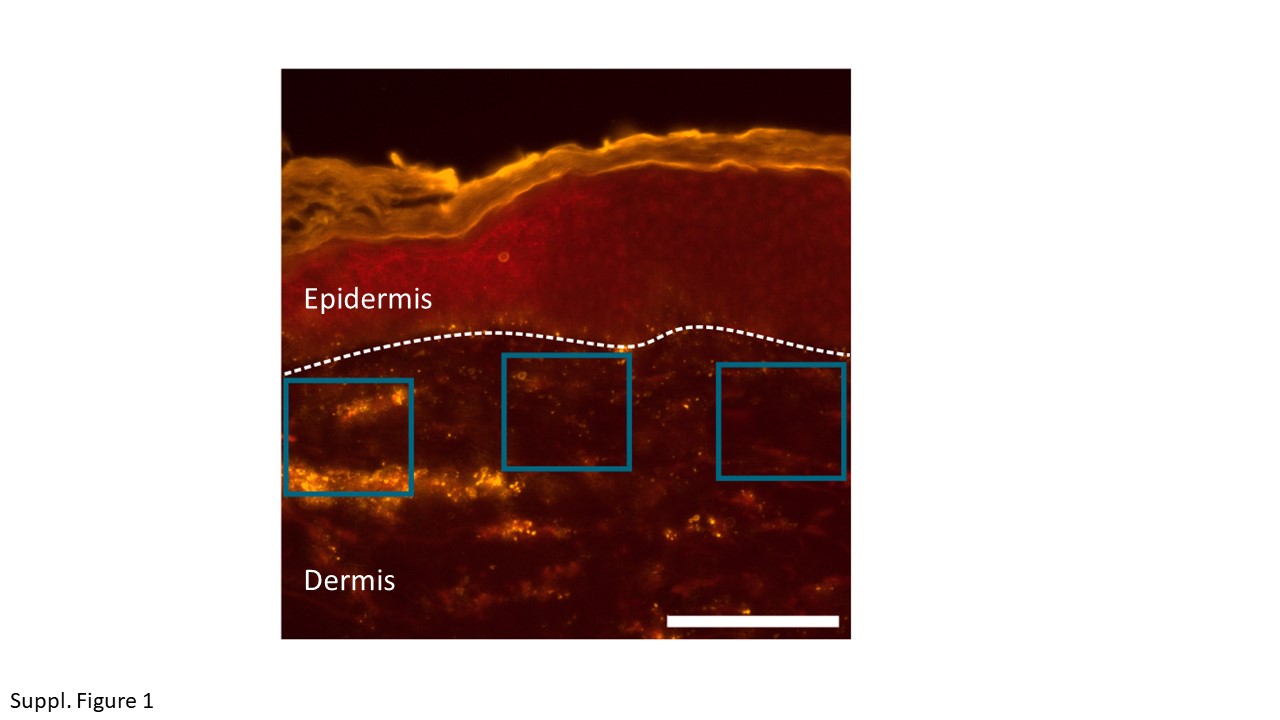

Supplement: Supplementary file 1 — Supplementary Material 1 [file 13023_2026_4490_MOESM1_ESM.jpg]

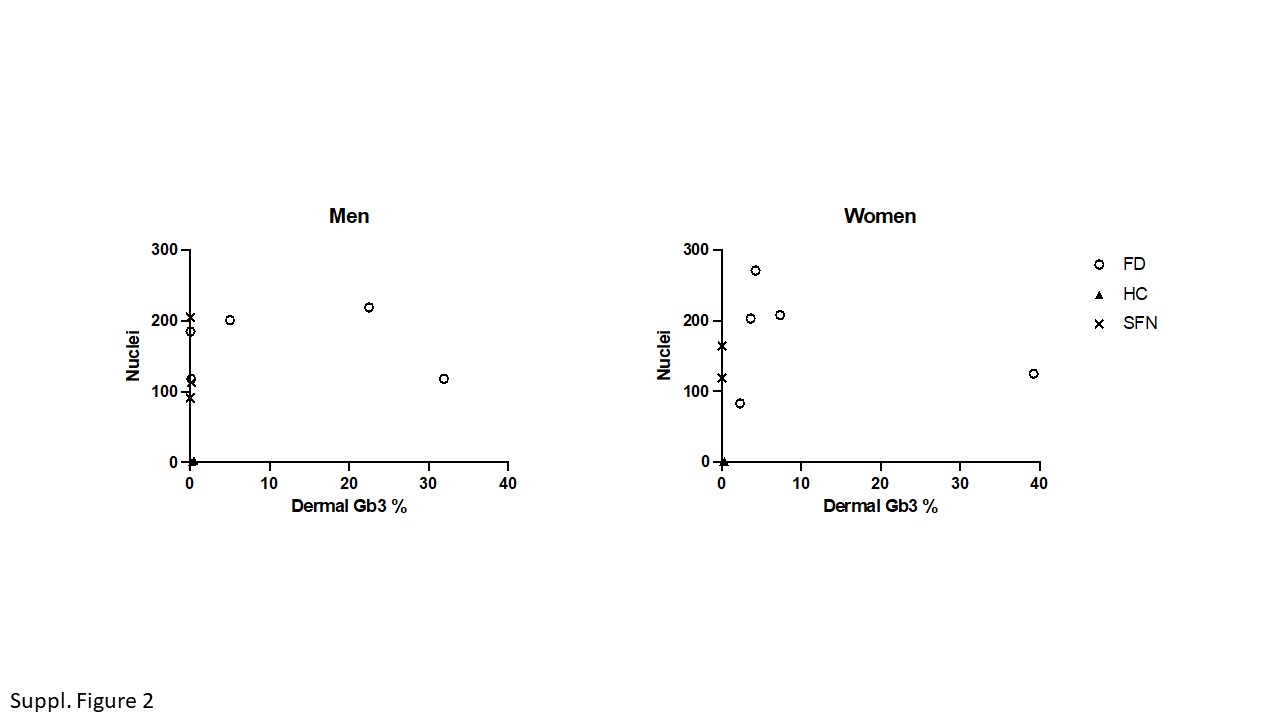

Supplement: Supplementary file 2 — Supplementary Material 2 [file 13023_2026_4490_MOESM2_ESM.jpg]
