# Supplementary material for: Automated quantification of skin Gb3 load and white matter lesion assessment in Fabry disease
Source: Orphanet J Rare Dis. 2026 Jul 7;21:245. doi: 10.1186/s13023-026-04490-4 (PMC13355356; doi:10.1186/s13023-026-04490-4)
Supplement: Supplementary file 3 — Supplementary Material 3 [file 13023_2026_4490_MOESM3_ESM.docx]

**Suppl. Table 2: Percentage of dermal Gb3 load in various patient groups with up to 11-years longterm follow up.**

| **Group allocation** | **Sex** | **Dermal Gb3 load [%]** | | | | | | | | | | | |
| --- | --- | --- | --- | --- | --- | --- | --- | --- | --- | --- | --- | --- | --- |
|  |  | **Baseline** | **Follow-up** | | | | | | | | | | |
|  |  |  | **Yr**  **1** | **Yr**  **2** | **Yr 3** | **Yr 4** | **Yr 5** | **Yr 6** | **Yr 7** | **Yr 8** | **Yr 9** | **Yr 10** | **Yr 11** |
| **Without Therapy** | | | | | | | | | | | | | |
| **G1** | M | 0 |  |  |  |  |  | 0 |  |  |  |  |  |
|  | F | 0 |  |  | 0 |  |  |  |  |  |  |  |  |
|  | F | 21 |  |  | 7 |  |  |  |  |  |  |  |  |
|  | F | 1 |  |  | 2 |  |  |  |  |  |  |  |  |
|  | F | 12 |  |  |  |  |  |  |  |  | 2 |  |  |
|  | F | 2 |  |  | 0 |  | 8 |  |  |  |  |  |  |
|  | F | 1 |  |  |  |  |  | 5 |  |  |  |  |  |
| **G2** | F | 0 |  | 1 |  |  |  |  |  |  |  |  |  |
|  | F | 1 |  |  |  |  | 1 |  |  |  |  |  |  |
|  | F | 0 |  | 3 |  |  |  |  |  |  |  |  |  |
|  | F | 0 |  |  | 3 |  |  |  |  |  | 0 |  |  |
| **G3** | M | 40 | 28 |  |  |  |  |  |  |  |  |  |  |
|  | F | 25 | 0 |  |  |  |  |  |  |  |  |  |  |
| **Constant therapy** | | | | | | | | | | | | | |
| **G1** | M | 0 |  |  |  | 28 |  | 10 |  |  |  |  |  |
|  | M | 12 | 24 |  |  |  |  |  |  |  |  |  |  |
|  | M | 8 |  |  | 7 |  |  |  |  |  |  |  |  |
|  | M | 9 |  |  | 36 |  |  | 24 |  | 15 | 8 |  |  |
|  | M | 7 | 2 |  |  |  |  |  |  |  |  |  |  |
| **G3** | M | 29 |  | 2 |  |  |  |  |  |  |  |  |  |
|  | F | 3 |  |  | 1 |  |  |  |  |  |  |  |  |
| **Therapy switch** | | | | | | | | | | | | | |
| **G1** | M | 22 | 3 | 2 |  |  | 5 |  |  |  |  |  |  |
|  | M | 2 |  |  |  |  |  |  |  | 1 |  |  |  |
|  | M | 6 | 0 |  |  |  |  |  |  |  |  |  |  |
|  | M | 21 |  | 24 |  | 8 |  | 16 |  |  |  |  |  |
|  | M | 3 |  |  | 15 |  |  |  |  |  |  |  |  |
|  | M | 0 |  |  |  | 0 |  |  |  |  |  |  |  |
|  | M | 3 |  |  |  |  |  |  |  | 21 |  |  |  |
|  | F | 25 |  | 1 |  |  |  |  |  |  |  |  |  |
|  | F | 1 | 0 |  |  |  |  |  |  |  |  |  |  |
|  | F | 4 |  |  | 2 |  |  | 5 |  |  |  |  |  |
|  | F | 1 |  | 5 |  |  |  |  |  |  |  |  |  |
|  | F | 3 | 8 |  |  |  |  |  |  |  |  |  |  |
| **G3** | F | 1 |  | 21 |  | 6 |  |  |  |  |  |  | 4 |

**Abbreviations:** F: female, M: male, Yr: year.

G1: pathogenic *GLA* variants, G2: non-pathogenic *GLA* variants, G3: variants of unknown significance.
