# Supplementary material for: Automated quantification of skin Gb3 load and white matter lesion assessment in Fabry disease
Source: Orphanet J Rare Dis. 2026 Jul 7;21:245. doi: 10.1186/s13023-026-04490-4 (PMC13355356; doi:10.1186/s13023-026-04490-4)
Supplement: Supplementary file 4 — Supplementary Material 4 [file 13023_2026_4490_MOESM4_ESM.docx]

**Supplementary Methods: CellProfiler pipeline for automated globotriaosylceramide (Gb3) quantification in human skin sections**

{

"has_image_plane_details": false,

"date_revision": 413,

"module_count": 14,

"modules": [

{

"attributes": {

"module_num": 1,

"notes": [

"To begin creating your project, use the Images module to compile a list of files and/or folders that you want to analyze. You can also specify a set of rules to include only the desired files in your selected folders."

],

"show_window": false,

"wants_pause": false,

"svn_version": "Unknown",

"enabled": true,

"variable_revision_number": 2,

"batch_state": "array([], dtype=uint8)",

"module_name": "Images",

"module_path": "cellprofiler_core.modules.images.Images"

},

"settings": [

{

"name": "cellprofiler_core.setting._path_list_display.PathListDisplay",

"text": "",

"value": ""

},

{

"name": "cellprofiler_core.setting.choice._choice.Choice",

"text": "Filter images?",

"value": "Images only"

},

{

"name": "cellprofiler_core.setting.filter._filter.Filter",

"text": "Select the rule criteria",

"value": "and (extension does isimage) (directory doesnot containregexp \"[\\\\\\\\/]\\\\.\")"

}

]

},

{

"attributes": {

"module_num": 2,

"notes": [

"The Metadata module optionally allows you to extract information describing your images (i.e, metadata) which will be stored along with your measurements. This information can be contained in the file name and/or location, or in an external file."

],

"show_window": false,

"wants_pause": false,

"svn_version": "Unknown",

"enabled": true,

"variable_revision_number": 6,

"batch_state": "array([], dtype=uint8)",

"module_name": "Metadata",

"module_path": "cellprofiler_core.modules.metadata.Metadata"

},

"settings": [

{

"name": "cellprofiler_core.setting._binary.Binary",

"text": "Extract metadata?",

"value": "No"

},

{

"name": "cellprofiler_core.setting.choice._choice.Choice",

"text": "Metadata data type",

"value": "Text"

},

{

"name": "cellprofiler_core.setting._data_types.DataTypes",

"text": "Metadata types",

"value": "{}"

},

{

"name": "cellprofiler_core.setting._hidden_count.HiddenCount",

"text": "Extraction method count",

"value": "1"

},

{

"name": "cellprofiler_core.setting.choice._choice.Choice",

"text": "Metadata extraction method",

"value": "Extract from image file headers"

},

{

"name": "cellprofiler_core.setting.choice._choice.Choice",

"text": "Metadata source",

"value": "File name"

},

{

"name": "cellprofiler_core.setting._regexp_text.RegexpText",

"text": "Regular expression to extract from file name",

"value": "^(?P<Plate>.*)_(?P<Well>[A-P][0-9]{2})_s(?P<Site>[0-9])_w(?P<ChannelNumber>[0-9])"

},

{

"name": "cellprofiler_core.setting._regexp_text.RegexpText",

"text": "Regular expression to extract from folder name",

"value": "(?P<Date>[0-9]{4}_[0-9]{2}_[0-9]{2})$"

},

{

"name": "cellprofiler_core.setting.choice._choice.Choice",

"text": "Extract metadata from",

"value": "All images"

},

{

"name": "cellprofiler_core.setting.filter._filter.Filter",

"text": "Select the filtering criteria",

"value": "and (file does contain \"\")"

},

{

"name": "cellprofiler_core.setting.text._directory.Directory",

"text": "Metadata file location",

"value": "Elsewhere...|"

},

{

"name": "cellprofiler_core.setting._joiner.Joiner",

"text": "Match file and image metadata",

"value": "[]"

},

{

"name": "cellprofiler_core.setting._binary.Binary",

"text": "Use case insensitive matching?",

"value": "No"

},

{

"name": "cellprofiler_core.setting.text._filename.Filename",

"text": "Metadata file name",

"value": ""

},

{

"name": "cellprofiler_core.setting._binary.Binary",

"text": "Does cached metadata exist?",

"value": "No"

}

]

},

{

"attributes": {

"module_num": 3,

"notes": [

"The NamesAndTypes module allows you to assign a meaningful name to each image by which other modules will refer to it.",

"\u2014",

"Load the images by matching files in the folder against the unique text pattern \u2018.JPG\u2019"

],

"show_window": false,

"wants_pause": false,

"svn_version": "Unknown",

"enabled": true,

"variable_revision_number": 8,

"batch_state": "array([], dtype=uint8)",

"module_name": "NamesAndTypes",

"module_path": "cellprofiler_core.modules.namesandtypes.NamesAndTypes"

},

"settings": [

{

"name": "cellprofiler_core.setting.choice._choice.Choice",

"text": "Assign a name to",

"value": "Images matching rules"

},

{

"name": "cellprofiler_core.setting.choice._choice.Choice",

"text": "Select the image type",

"value": "Grayscale image"

},

{

"name": "cellprofiler_core.setting.text.alphanumeric.name.image_name._file_image_name.FileImageName",

"text": "Name to assign these images",

"value": "DNA"

},

{

"name": "cellprofiler_core.setting._joiner.Joiner",

"text": "Match metadata",

"value": "[]"

},

{

"name": "cellprofiler_core.setting.choice._choice.Choice",

"text": "Image set matching method",

"value": "Order"

},

{

"name": "cellprofiler_core.setting.choice._choice.Choice",

"text": "Set intensity range from",

"value": "Image metadata"

},

{

"name": "cellprofiler_core.setting._hidden_count.HiddenCount",

"text": "Assignments count",

"value": "2"

},

{

"name": "cellprofiler_core.setting._hidden_count.HiddenCount",

"text": "Single images count",

"value": "0"

},

{

"name": "cellprofiler_core.setting.text.number._float.Float",

"text": "Maximum intensity",

"value": "255.0"

},

{

"name": "cellprofiler_core.setting._binary.Binary",

"text": "Process as 3D?",

"value": "No"

},

{

"name": "cellprofiler_core.setting.text.number._float.Float",

"text": "Relative pixel spacing in X",

"value": "1.0"

},

{

"name": "cellprofiler_core.setting.text.number._float.Float",

"text": "Relative pixel spacing in Y",

"value": "1.0"

},

{

"name": "cellprofiler_core.setting.text.number._float.Float",

"text": "Relative pixel spacing in Z",

"value": "1.0"

},

{

"name": "cellprofiler_core.setting.filter._filter.Filter",

"text": "Select the rule criteria",

"value": "and (file does contain \"Gb3\")"

},

{

"name": "cellprofiler_core.setting.text.alphanumeric.name.image_name._file_image_name.FileImageName",

"text": "Name to assign these images",

"value": "Gb3"

},

{

"name": "cellprofiler_core.setting.text.alphanumeric.name._label_name.LabelName",

"text": "Name to assign these objects",

"value": "Cell"

},

{

"name": "cellprofiler_core.setting.choice._choice.Choice",

"text": "Select the image type",

"value": "Grayscale image"

},

{

"name": "cellprofiler_core.setting.choice._choice.Choice",

"text": "Set intensity range from",

"value": "Image metadata"

},

{

"name": "cellprofiler_core.setting.text.number._float.Float",

"text": "Maximum intensity",

"value": "255.0"

},

{

"name": "cellprofiler_core.setting.filter._filter.Filter",

"text": "Select the rule criteria",

"value": "and (file does contain \"DAPI\")"

},

{

"name": "cellprofiler_core.setting.text.alphanumeric.name.image_name._file_image_name.FileImageName",

"text": "Name to assign these images",

"value": "DAPI"

},

{

"name": "cellprofiler_core.setting.text.alphanumeric.name._label_name.LabelName",

"text": "Name to assign these objects",

"value": "Nucleus"

},

{

"name": "cellprofiler_core.setting.choice._choice.Choice",

"text": "Select the image type",

"value": "Grayscale image"

},

{

"name": "cellprofiler_core.setting.choice._choice.Choice",

"text": "Set intensity range from",

"value": "Image metadata"

},

{

"name": "cellprofiler_core.setting.text.number._float.Float",

"text": "Maximum intensity",

"value": "255.0"

}

]

},

{

"attributes": {

"module_num": 4,

"notes": [

"The Groups module optionally allows you to split your list of images into image subsets (groups) which will be processed independently of each other. Examples of groupings include screening batches, microtiter plates, time-lapse movies, etc."

],

"show_window": false,

"wants_pause": false,

"svn_version": "Unknown",

"enabled": true,

"variable_revision_number": 2,

"batch_state": "array([], dtype=uint8)",

"module_name": "Groups",

"module_path": "cellprofiler_core.modules.groups.Groups"

},

"settings": [

{

"name": "cellprofiler_core.setting._binary.Binary",

"text": "Do you want to group your images?",

"value": "No"

},

{

"name": "cellprofiler_core.setting._hidden_count.HiddenCount",

"text": "grouping metadata count",

"value": "1"

},

{

"name": "cellprofiler_core.setting.choice._choice.Choice",

"text": "Metadata category",

"value": "None"

}

]

},

{

"attributes": {

"module_num": 5,

"notes": [

"Combine the color image into a grayscale image."

],

"show_window": false,

"wants_pause": false,

"svn_version": "Unknown",

"enabled": false,

"variable_revision_number": 4,

"batch_state": "array([], dtype=uint8)",

"module_name": "ColorToGray",

"module_path": "cellprofiler.modules.colortogray.ColorToGray"

},

"settings": [

{

"name": "cellprofiler_core.setting.subscriber.image_subscriber._image_subscriber.ImageSubscriber",

"text": "Select the input image",

"value": "OrigColor"

},

{

"name": "cellprofiler_core.setting.choice._choice.Choice",

"text": "Conversion method",

"value": "Combine"

},

{

"name": "cellprofiler_core.setting.choice._choice.Choice",

"text": "Image type",

"value": "RGB"

},

{

"name": "cellprofiler_core.setting.text.alphanumeric.name.image_name._image_name.ImageName",

"text": "Name the output image",

"value": "OrigGray"

},

{

"name": "cellprofiler_core.setting.text.number._float.Float",

"text": "Relative weight of the red channel",

"value": "1.0"

},

{

"name": "cellprofiler_core.setting.text.number._float.Float",

"text": "Relative weight of the green channel",

"value": "1.0"

},

{

"name": "cellprofiler_core.setting.text.number._float.Float",

"text": "Relative weight of the blue channel",

"value": "1.0"

},

{

"name": "cellprofiler_core.setting._binary.Binary",

"text": "Convert red to gray?",

"value": "Yes"

},

{

"name": "cellprofiler_core.setting.text.alphanumeric.name.image_name._image_name.ImageName",

"text": "Name the output image",

"value": "OrigRed"

},

{

"name": "cellprofiler_core.setting._binary.Binary",

"text": "Convert green to gray?",

"value": "Yes"

},

{

"name": "cellprofiler_core.setting.text.alphanumeric.name.image_name._image_name.ImageName",

"text": "Name the output image",

"value": "OrigGreen"

},

{

"name": "cellprofiler_core.setting._binary.Binary",

"text": "Convert blue to gray?",

"value": "Yes"

},

{

"name": "cellprofiler_core.setting.text.alphanumeric.name.image_name._image_name.ImageName",

"text": "Name the output image",

"value": "OrigBlue"

},

{

"name": "cellprofiler_core.setting._binary.Binary",

"text": "Convert hue to gray?",

"value": "Yes"

},

{

"name": "cellprofiler_core.setting.text.alphanumeric.name.image_name._image_name.ImageName",

"text": "Name the output image",

"value": "OrigHue"

},

{

"name": "cellprofiler_core.setting._binary.Binary",

"text": "Convert saturation to gray?",

"value": "Yes"

},

{

"name": "cellprofiler_core.setting.text.alphanumeric.name.image_name._image_name.ImageName",

"text": "Name the output image",

"value": "OrigSaturation"

},

{

"name": "cellprofiler_core.setting._binary.Binary",

"text": "Convert value to gray?",

"value": "Yes"

},

{

"name": "cellprofiler_core.setting.text.alphanumeric.name.image_name._image_name.ImageName",

"text": "Name the output image",

"value": "OrigValue"

},

{

"name": "cellprofiler_core.setting._hidden_count.HiddenCount",

"text": "Channel count",

"value": "1"

},

{

"name": "cellprofiler_core.setting.text.number.integer._integer.Integer",

"text": "Channel number",

"value": "1"

},

{

"name": "cellprofiler_core.setting.text.number._float.Float",

"text": "Relative weight of the channel",

"value": "1.0"

},

{

"name": "cellprofiler_core.setting.text.alphanumeric.name.image_name._image_name.ImageName",

"text": "Image name",

"value": "Channel1"

}

]

},

{

"attributes": {

"module_num": 6,

"notes": [

"Smooth the image using a Gaussian filter."

],

"show_window": false,

"wants_pause": false,

"svn_version": "Unknown",

"enabled": false,

"variable_revision_number": 2,

"batch_state": "array([], dtype=uint8)",

"module_name": "Smooth",

"module_path": "cellprofiler.modules.smooth.Smooth"

},

"settings": [

{

"name": "cellprofiler_core.setting.subscriber.image_subscriber._image_subscriber.ImageSubscriber",

"text": "Select the input image",

"value": "Gb3"

},

{

"name": "cellprofiler_core.setting.text.alphanumeric.name.image_name._image_name.ImageName",

"text": "Name the output image",

"value": "Corrected"

},

{

"name": "cellprofiler_core.setting.choice._choice.Choice",

"text": "Select smoothing method",

"value": "Gaussian Filter"

},

{

"name": "cellprofiler_core.setting._binary.Binary",

"text": "Calculate artifact diameter automatically?",

"value": "No"

},

{

"name": "cellprofiler_core.setting.text.number._float.Float",

"text": "Typical artifact diameter",

"value": "20"

},

{

"name": "cellprofiler_core.setting.text.number._float.Float",

"text": "Edge intensity difference",

"value": "0.1"

},

{

"name": "cellprofiler_core.setting._binary.Binary",

"text": "Clip intensities to 0 and 1?",

"value": "Yes"

}

]

},

{

"attributes": {

"module_num": 7,

"notes": [

"Identify the tissue region using three-class Otsu."

],

"show_window": true,

"wants_pause": false,

"svn_version": "Unknown",

"enabled": true,

"variable_revision_number": 14,

"batch_state": "array([], dtype=uint8)",

"module_name": "IdentifyPrimaryObjects",

"module_path": "cellprofiler.modules.identifyprimaryobjects.IdentifyPrimaryObjects"

},

"settings": [

{

"name": "cellprofiler_core.setting.subscriber.image_subscriber._image_subscriber.ImageSubscriber",

"text": "Select the input image",

"value": "Gb3"

},

{

"name": "cellprofiler_core.setting.text.alphanumeric.name._label_name.LabelName",

"text": "Name the primary objects to be identified",

"value": "Tissue"

},

{

"name": "cellprofiler_core.setting.range.integer_range._integer_range.IntegerRange",

"text": "Typical diameter of objects, in pixel units (Min,Max)",

"value": "1,2"

},

{

"name": "cellprofiler_core.setting._binary.Binary",

"text": "Discard objects outside the diameter range?",

"value": "No"

},

{

"name": "cellprofiler_core.setting._binary.Binary",

"text": "Discard objects touching the border of the image?",

"value": "No"

},

{

"name": "cellprofiler_core.setting.choice._choice.Choice",

"text": "Method to distinguish clumped objects",

"value": "None"

},

{

"name": "cellprofiler_core.setting.choice._choice.Choice",

"text": "Method to draw dividing lines between clumped objects",

"value": "Intensity"

},

{

"name": "cellprofiler_core.setting.text.number.integer._integer.Integer",

"text": "Size of smoothing filter",

"value": "10"

},

{

"name": "cellprofiler_core.setting.text.number._float.Float",

"text": "Suppress local maxima that are closer than this minimum allowed distance",

"value": "7.0"

},

{

"name": "cellprofiler_core.setting._binary.Binary",

"text": "Speed up by using lower-resolution image to find local maxima?",

"value": "Yes"

},

{

"name": "cellprofiler_core.setting.choice._choice.Choice",

"text": "Fill holes in identified objects?",

"value": "Never"

},

{

"name": "cellprofiler_core.setting._binary.Binary",

"text": "Automatically calculate size of smoothing filter for declumping?",

"value": "Yes"

},

{

"name": "cellprofiler_core.setting._binary.Binary",

"text": "Automatically calculate minimum allowed distance between local maxima?",

"value": "Yes"

},

{

"name": "cellprofiler_core.setting.choice._choice.Choice",

"text": "Handling of objects if excessive number of objects identified",

"value": "Continue"

},

{

"name": "cellprofiler_core.setting.text.number.integer._integer.Integer",

"text": "Maximum number of objects",

"value": "500"

},

{

"name": "cellprofiler_core.setting._binary.Binary",

"text": "Display accepted local maxima?",

"value": "No"

},

{

"name": "cellprofiler_core.setting._color.Color",

"text": "Select maxima color",

"value": "Blue"

},

{

"name": "cellprofiler_core.setting._binary.Binary",

"text": "Use advanced settings?",

"value": "Yes"

},

{

"name": "cellprofiler_core.setting.text.number.integer._integer.Integer",

"text": "Threshold setting version",

"value": "12"

},

{

"name": "cellprofiler_core.setting.choice._choice.Choice",

"text": "Threshold strategy",

"value": "Global"

},

{

"name": "cellprofiler_core.setting.choice._choice.Choice",

"text": "Thresholding method",

"value": "Otsu"

},

{

"name": "cellprofiler_core.setting.text.number._float.Float",

"text": "Threshold smoothing scale",

"value": "1.3488"

},

{

"name": "cellprofiler_core.setting.text.number._float.Float",

"text": "Threshold correction factor",

"value": "1.2"

},

{

"name": "cellprofiler_core.setting.range._float_range.FloatRange",

"text": "Lower and upper bounds on threshold",

"value": "0.025,1.0"

},

{

"name": "cellprofiler_core.setting.text.number._float.Float",

"text": "Manual threshold",

"value": "0.0"

},

{

"name": "cellprofiler_core.setting._measurement.Measurement",

"text": "Select the measurement to threshold with",

"value": "None"

},

{

"name": "cellprofiler_core.setting.choice._choice.Choice",

"text": "Two-class or three-class thresholding?",

"value": "Three classes"

},

{

"name": "cellprofiler_core.setting._binary.Binary",

"text": "Log transform before thresholding?",

"value": "Yes"

},

{

"name": "cellprofiler_core.setting.choice._choice.Choice",

"text": "Assign pixels in the middle intensity class to the foreground or the background?",

"value": "Foreground"

},

{

"name": "cellprofiler_core.setting.text.number.integer._integer.Integer",

"text": "Size of adaptive window",

"value": "50"

},

{

"name": "cellprofiler_core.setting.text.number._float.Float",

"text": "Lower outlier fraction",

"value": "0.05"

},

{

"name": "cellprofiler_core.setting.text.number._float.Float",

"text": "Upper outlier fraction",

"value": "0.05"

},

{

"name": "cellprofiler_core.setting.choice._choice.Choice",

"text": "Averaging method",

"value": "Mean"

},

{

"name": "cellprofiler_core.setting.choice._choice.Choice",

"text": "Variance method",

"value": "Standard deviation"

},

{

"name": "cellprofiler_core.setting.text.number._float.Float",

"text": "# of deviations",

"value": "2.0"

},

{

"name": "cellprofiler_core.setting.choice._choice.Choice",

"text": "Thresholding method",

"value": "Otsu"

}

]

},

{

"attributes": {

"module_num": 8,

"notes": [],

"show_window": true,

"wants_pause": false,

"svn_version": "Unknown",

"enabled": true,

"variable_revision_number": 4,

"batch_state": "array([], dtype=uint8)",

"module_name": "OverlayOutlines",

"module_path": "cellprofiler.modules.overlayoutlines.OverlayOutlines"

},

"settings": [

{

"name": "cellprofiler_core.setting._binary.Binary",

"text": "Display outlines on a blank image?",

"value": "No"

},

{

"name": "cellprofiler_core.setting.subscriber.image_subscriber._image_subscriber.ImageSubscriber",

"text": "Select image on which to display outlines",

"value": "Gb3"

},

{

"name": "cellprofiler_core.setting.text.alphanumeric.name.image_name._image_name.ImageName",

"text": "Name the output image",

"value": "OrigOverlay"

},

{

"name": "cellprofiler_core.setting.choice._choice.Choice",

"text": "Outline display mode",

"value": "Color"

},

{

"name": "cellprofiler_core.setting.choice._choice.Choice",

"text": "Select method to determine brightness of outlines",

"value": "Max of image"

},

{

"name": "cellprofiler_core.setting.choice._choice.Choice",

"text": "How to outline",

"value": "Inner"

},

{

"name": "cellprofiler_core.setting._color.Color",

"text": "Select outline color",

"value": "Red"

},

{

"name": "cellprofiler_core.setting.subscriber._label_subscriber.LabelSubscriber",

"text": "Select objects to display",

"value": "Tissue"

}

]

},

{

"attributes": {

"module_num": 9,

"notes": [],

"show_window": false,

"wants_pause": false,

"svn_version": "Unknown",

"enabled": true,

"variable_revision_number": 14,

"batch_state": "array([], dtype=uint8)",

"module_name": "IdentifyPrimaryObjects",

"module_path": "cellprofiler.modules.identifyprimaryobjects.IdentifyPrimaryObjects"

},

"settings": [

{

"name": "cellprofiler_core.setting.subscriber.image_subscriber._image_subscriber.ImageSubscriber",

"text": "Select the input image",

"value": "DAPI"

},

{

"name": "cellprofiler_core.setting.text.alphanumeric.name._label_name.LabelName",

"text": "Name the primary objects to be identified",

"value": "Nuclei"

},

{

"name": "cellprofiler_core.setting.range.integer_range._integer_range.IntegerRange",

"text": "Typical diameter of objects, in pixel units (Min,Max)",

"value": "20,120"

},

{

"name": "cellprofiler_core.setting._binary.Binary",

"text": "Discard objects outside the diameter range?",

"value": "Yes"

},

{

"name": "cellprofiler_core.setting._binary.Binary",

"text": "Discard objects touching the border of the image?",

"value": "No"

},

{

"name": "cellprofiler_core.setting.choice._choice.Choice",

"text": "Method to distinguish clumped objects",

"value": "Intensity"

},

{

"name": "cellprofiler_core.setting.choice._choice.Choice",

"text": "Method to draw dividing lines between clumped objects",

"value": "Intensity"

},

{

"name": "cellprofiler_core.setting.text.number.integer._integer.Integer",

"text": "Size of smoothing filter",

"value": "10"

},

{

"name": "cellprofiler_core.setting.text.number._float.Float",

"text": "Suppress local maxima that are closer than this minimum allowed distance",

"value": "7.0"

},

{

"name": "cellprofiler_core.setting._binary.Binary",

"text": "Speed up by using lower-resolution image to find local maxima?",

"value": "Yes"

},

{

"name": "cellprofiler_core.setting.choice._choice.Choice",

"text": "Fill holes in identified objects?",

"value": "Never"

},

{

"name": "cellprofiler_core.setting._binary.Binary",

"text": "Automatically calculate size of smoothing filter for declumping?",

"value": "Yes"

},

{

"name": "cellprofiler_core.setting._binary.Binary",

"text": "Automatically calculate minimum allowed distance between local maxima?",

"value": "Yes"

},

{

"name": "cellprofiler_core.setting.choice._choice.Choice",

"text": "Handling of objects if excessive number of objects identified",

"value": "Continue"

},

{

"name": "cellprofiler_core.setting.text.number.integer._integer.Integer",

"text": "Maximum number of objects",

"value": "500"

},

{

"name": "cellprofiler_core.setting._binary.Binary",

"text": "Display accepted local maxima?",

"value": "No"

},

{

"name": "cellprofiler_core.setting._color.Color",

"text": "Select maxima color",

"value": "Blue"

},

{

"name": "cellprofiler_core.setting._binary.Binary",

"text": "Use advanced settings?",

"value": "Yes"

},

{

"name": "cellprofiler_core.setting.text.number.integer._integer.Integer",

"text": "Threshold setting version",

"value": "12"

},

{

"name": "cellprofiler_core.setting.choice._choice.Choice",

"text": "Threshold strategy",

"value": "Global"

},

{

"name": "cellprofiler_core.setting.choice._choice.Choice",

"text": "Thresholding method",

"value": "Otsu"

},

{

"name": "cellprofiler_core.setting.text.number._float.Float",

"text": "Threshold smoothing scale",

"value": "1.3488"

},

{

"name": "cellprofiler_core.setting.text.number._float.Float",

"text": "Threshold correction factor",

"value": "1.0"

},

{

"name": "cellprofiler_core.setting.range._float_range.FloatRange",

"text": "Lower and upper bounds on threshold",

"value": "0.05,1.0"

},

{

"name": "cellprofiler_core.setting.text.number._float.Float",

"text": "Manual threshold",

"value": "0.0"

},

{

"name": "cellprofiler_core.setting._measurement.Measurement",

"text": "Select the measurement to threshold with",

"value": "None"

},

{

"name": "cellprofiler_core.setting.choice._choice.Choice",

"text": "Two-class or three-class thresholding?",

"value": "Three classes"

},

{

"name": "cellprofiler_core.setting._binary.Binary",

"text": "Log transform before thresholding?",

"value": "Yes"

},

{

"name": "cellprofiler_core.setting.choice._choice.Choice",

"text": "Assign pixels in the middle intensity class to the foreground or the background?",

"value": "Background"

},

{

"name": "cellprofiler_core.setting.text.number.integer._integer.Integer",

"text": "Size of adaptive window",

"value": "50"

},

{

"name": "cellprofiler_core.setting.text.number._float.Float",

"text": "Lower outlier fraction",

"value": "0.05"

},

{

"name": "cellprofiler_core.setting.text.number._float.Float",

"text": "Upper outlier fraction",

"value": "0.05"

},

{

"name": "cellprofiler_core.setting.choice._choice.Choice",

"text": "Averaging method",

"value": "Mean"

},

{

"name": "cellprofiler_core.setting.choice._choice.Choice",

"text": "Variance method",

"value": "Standard deviation"

},

{

"name": "cellprofiler_core.setting.text.number._float.Float",

"text": "# of deviations",

"value": "2.0"

},

{

"name": "cellprofiler_core.setting.choice._choice.Choice",

"text": "Thresholding method",

"value": "Otsu"

}

]

},

{

"attributes": {

"module_num": 10,

"notes": [

"Measure the area occupied by the tissue region."

],

"show_window": false,

"wants_pause": false,

"svn_version": "Unknown",

"enabled": true,

"variable_revision_number": 5,

"batch_state": "array([], dtype=uint8)",

"module_name": "MeasureImageAreaOccupied",

"module_path": "cellprofiler.modules.measureimageareaoccupied.MeasureImageAreaOccupied"

},

"settings": [

{

"name": "cellprofiler_core.setting.choice._choice.Choice",

"text": "Measure the area occupied by",

"value": "Objects"

},

{

"name": "cellprofiler_core.setting.subscriber.list_subscriber._image_list_subscriber.ImageListSubscriber",

"text": "Select binary images to measure",

"value": ""

},

{

"name": "cellprofiler_core.setting.subscriber.list_subscriber._label_list_subscriber.LabelListSubscriber",

"text": "Select object sets to measure",

"value": "Tissue"

}

]

},

{

"attributes": {

"module_num": 11,

"notes": [

"Gb3 prozentual auf Gesamtfl\u00c3\u00a4che bezogen"

],

"show_window": false,

"wants_pause": false,

"svn_version": "Unknown",

"enabled": true,

"variable_revision_number": 3,

"batch_state": "array([], dtype=uint8)",

"module_name": "CalculateMath",

"module_path": "cellprofiler.modules.calculatemath.CalculateMath"

},

"settings": [

{

"name": "cellprofiler_core.setting.text.alphanumeric._alphanumeric.Alphanumeric",

"text": "Name the output measurement",

"value": "PercentArea"

},

{

"name": "cellprofiler_core.setting.choice._choice.Choice",

"text": "Operation",

"value": "Divide"

},

{

"name": "cellprofiler_core.setting.choice._choice.Choice",

"text": "Select the numerator measurement type",

"value": "Image"

},

{

"name": "cellprofiler_core.setting.subscriber._label_subscriber.LabelSubscriber",

"text": "Select the numerator objects",

"value": "Tissue"

},

{

"name": "cellprofiler_core.setting._measurement.Measurement",

"text": "Select the numerator measurement",

"value": "AreaOccupied_AreaOccupied_Tissue"

},

{

"name": "cellprofiler_core.setting.text.number._float.Float",

"text": "Multiply the above operand by",

"value": "100"

},

{

"name": "cellprofiler_core.setting.text.number._float.Float",

"text": "Raise the power of above operand by",

"value": "1.0"

},

{

"name": "cellprofiler_core.setting.choice._choice.Choice",

"text": "Select the denominator measurement type",

"value": "Image"

},

{

"name": "cellprofiler_core.setting.subscriber._label_subscriber.LabelSubscriber",

"text": "Select the denominator objects",

"value": "None"

},

{

"name": "cellprofiler_core.setting._measurement.Measurement",

"text": "Select the denominator measurement",

"value": "AreaOccupied_TotalArea_Tissue"

},

{

"name": "cellprofiler_core.setting.text.number._float.Float",

"text": "Multiply the above operand by",

"value": "1.0"

},

{

"name": "cellprofiler_core.setting.text.number._float.Float",

"text": "Raise the power of above operand by",

"value": "1.0"

},

{

"name": "cellprofiler_core.setting._binary.Binary",

"text": "Take log10 of result?",

"value": "No"

},

{

"name": "cellprofiler_core.setting.text.number._float.Float",

"text": "Multiply the result by",

"value": "1.0"

},

{

"name": "cellprofiler_core.setting.text.number._float.Float",

"text": "Raise the power of result by",

"value": "1.0"

},

{

"name": "cellprofiler_core.setting.text.number._float.Float",

"text": "Add to the result",

"value": "0.0"

},

{

"name": "cellprofiler_core.setting.choice._choice.Choice",

"text": "How should the output value be rounded?",

"value": "Not rounded"

},

{

"name": "cellprofiler_core.setting.text.number.integer._integer.Integer",

"text": "Enter how many decimal places the value should be rounded to",

"value": "0"

},

{

"name": "cellprofiler_core.setting._binary.Binary",

"text": "Constrain the result to a lower bound?",

"value": "No"

},

{

"name": "cellprofiler_core.setting.text.number._float.Float",

"text": "Enter the lower bound",

"value": "0.0"

},

{

"name": "cellprofiler_core.setting._binary.Binary",

"text": "Constrain the result to an upper bound?",

"value": "No"

},

{

"name": "cellprofiler_core.setting.text.number._float.Float",

"text": "Enter the upper bound",

"value": "1.0"

}

]

},

{

"attributes": {

"module_num": 12,

"notes": [

"Gb3 \"pro\" Zelle;",

"Fl\u00c3\u00a4che Gb3 / Anzahl Nuclei",

"0,01288 = Skalierungsfaktor von px\u00c2\u00b2 zu \u00c2\u00b5m\u00c2\u00b2"

],

"show_window": false,

"wants_pause": false,

"svn_version": "Unknown",

"enabled": true,

"variable_revision_number": 3,

"batch_state": "array([], dtype=uint8)",

"module_name": "CalculateMath",

"module_path": "cellprofiler.modules.calculatemath.CalculateMath"

},

"settings": [

{

"name": "cellprofiler_core.setting.text.alphanumeric._alphanumeric.Alphanumeric",

"text": "Name the output measurement",

"value": "Gb3Cell"

},

{

"name": "cellprofiler_core.setting.choice._choice.Choice",

"text": "Operation",

"value": "Divide"

},

{

"name": "cellprofiler_core.setting.choice._choice.Choice",

"text": "Select the numerator measurement type",

"value": "Image"

},

{

"name": "cellprofiler_core.setting.subscriber._label_subscriber.LabelSubscriber",

"text": "Select the numerator objects",

"value": "None"

},

{

"name": "cellprofiler_core.setting._measurement.Measurement",

"text": "Select the numerator measurement",

"value": "AreaOccupied_AreaOccupied_Tissue"

},

{

"name": "cellprofiler_core.setting.text.number._float.Float",

"text": "Multiply the above operand by",

"value": "0.01288"

},

{

"name": "cellprofiler_core.setting.text.number._float.Float",

"text": "Raise the power of above operand by",

"value": "1.0"

},

{

"name": "cellprofiler_core.setting.choice._choice.Choice",

"text": "Select the denominator measurement type",

"value": "Image"

},

{

"name": "cellprofiler_core.setting.subscriber._label_subscriber.LabelSubscriber",

"text": "Select the denominator objects",

"value": "None"

},

{

"name": "cellprofiler_core.setting._measurement.Measurement",

"text": "Select the denominator measurement",

"value": "Count_Nuclei"

},

{

"name": "cellprofiler_core.setting.text.number._float.Float",

"text": "Multiply the above operand by",

"value": "1.0"

},

{

"name": "cellprofiler_core.setting.text.number._float.Float",

"text": "Raise the power of above operand by",

"value": "1.0"

},

{

"name": "cellprofiler_core.setting._binary.Binary",

"text": "Take log10 of result?",

"value": "No"

},

{

"name": "cellprofiler_core.setting.text.number._float.Float",

"text": "Multiply the result by",

"value": "1.0"

},

{

"name": "cellprofiler_core.setting.text.number._float.Float",

"text": "Raise the power of result by",

"value": "1.0"

},

{

"name": "cellprofiler_core.setting.text.number._float.Float",

"text": "Add to the result",

"value": "0.0"

},

{

"name": "cellprofiler_core.setting.choice._choice.Choice",

"text": "How should the output value be rounded?",

"value": "Not rounded"

},

{

"name": "cellprofiler_core.setting.text.number.integer._integer.Integer",

"text": "Enter how many decimal places the value should be rounded to",

"value": "0"

},

{

"name": "cellprofiler_core.setting._binary.Binary",

"text": "Constrain the result to a lower bound?",

"value": "No"

},

{

"name": "cellprofiler_core.setting.text.number._float.Float",

"text": "Enter the lower bound",

"value": "0.0"

},

{

"name": "cellprofiler_core.setting._binary.Binary",

"text": "Constrain the result to an upper bound?",

"value": "No"

},

{

"name": "cellprofiler_core.setting.text.number._float.Float",

"text": "Enter the upper bound",

"value": "1.0"

}

]

},

{

"attributes": {

"module_num": 13,

"notes": [

"Export any measurements to a comma-delimited file (.csv). Since the tissue area is an image measurement, it is included in the per-image file."

],

"show_window": false,

"wants_pause": false,

"svn_version": "Unknown",

"enabled": true,

"variable_revision_number": 13,

"batch_state": "array([], dtype=uint8)",

"module_name": "ExportToSpreadsheet",

"module_path": "cellprofiler.modules.exporttospreadsheet.ExportToSpreadsheet"

},

"settings": [

{

"name": "cellprofiler_core.setting.choice._custom_choice.CustomChoice",

"text": "Select the column delimiter",

"value": "Tab"

},

{

"name": "cellprofiler_core.setting._binary.Binary",

"text": "Add image metadata columns to your object data file?",

"value": "No"

},

{

"name": "cellprofiler_core.setting._binary.Binary",

"text": "Add image file and folder names to your object data file?",

"value": "No"

},

{

"name": "cellprofiler_core.setting._binary.Binary",

"text": "Select the measurements to export",

"value": "Yes"

},

{

"name": "cellprofiler_core.setting._binary.Binary",

"text": "Calculate the per-image mean values for object measurements?",

"value": "Yes"

},

{

"name": "cellprofiler_core.setting._binary.Binary",

"text": "Calculate the per-image median values for object measurements?",

"value": "No"

},

{

"name": "cellprofiler_core.setting._binary.Binary",

"text": "Calculate the per-image standard deviation values for object measurements?",

"value": "No"

},

{

"name": "cellprofiler_core.setting.text._directory.Directory",

"text": "Output file location",

"value": "Default Input Folder sub-folder|Documents\\\\Gb3_Pfister\\\\Output"

},

{

"name": "cellprofiler_core.setting._binary.Binary",

"text": "Create a GenePattern GCT file?",

"value": "No"

},

{

"name": "cellprofiler_core.setting.choice._choice.Choice",

"text": "Select source of sample row name",

"value": "Metadata"

},

{

"name": "cellprofiler_core.setting.subscriber.image_subscriber._image_subscriber.ImageSubscriber",

"text": "Select the image to use as the identifier",

"value": "None"

},

{

"name": "cellprofiler_core.setting._measurement.Measurement",

"text": "Select the metadata to use as the identifier",

"value": "None"

},

{

"name": "cellprofiler_core.setting._binary.Binary",

"text": "Export all measurement types?",

"value": "No"

},

{

"name": "cellprofiler_core.setting.multichoice._measurement_multichoice.MeasurementMultiChoice",

"text": "Press button to select measurements",

"value": "Image|Count_Nuclei,Image|Count_Tissue,Image|AreaOccupied_AreaOccupied_Tissue,Image|AreaOccupied_TotalArea_Tissue,Image|ModuleError_02Metadata,Image|ModuleError_04Groups,Image|ModuleError_07IdentifyPrimaryObjects,Image|ModuleError_03NamesAndTypes,Image|ModuleError_01Images,Image|Threshold_FinalThreshold_Tissue,Image|Threshold_OrigThreshold_Tissue,Image|Threshold_SumOfEntropies_Tissue,Image|Threshold_WeightedVariance_Tissue,Image|FileName_DAPI,Image|Math_Gb3Cell,Image|Math_PercentArea"

},

{

"name": "cellprofiler_core.setting.choice._choice.Choice",

"text": "Representation of Nan/Inf",

"value": "NaN"

},

{

"name": "cellprofiler_core.setting._binary.Binary",

"text": "Add a prefix to file names?",

"value": "No"

},

{

"name": "cellprofiler_core.setting.text._text.Text",

"text": "Filename prefix",

"value": "MyExpt_"

},

{

"name": "cellprofiler_core.setting._binary.Binary",

"text": "Overwrite existing files without warning?",

"value": "Yes"

},

{

"name": "cellprofiler.modules.exporttospreadsheet.EEObjectNameSubscriber",

"text": "Data to export",

"value": "Image"

},

{

"name": "cellprofiler_core.setting._binary.Binary",

"text": "Combine these object measurements with those of the previous object?",

"value": "No"

},

{

"name": "cellprofiler_core.setting.text._text.Text",

"text": "File name",

"value": "DATA.csv"

},

{

"name": "cellprofiler_core.setting._binary.Binary",

"text": "Use the object name for the file name?",

"value": "Yes"

}

]

},

{

"attributes": {

"module_num": 14,

"notes": [],

"show_window": true,

"wants_pause": false,

"svn_version": "Unknown",

"enabled": true,

"variable_revision_number": 15,

"batch_state": "array([], dtype=uint8)",

"module_name": "SaveImages",

"module_path": "cellprofiler.modules.saveimages.SaveImages"

},

"settings": [

{

"name": "cellprofiler_core.setting.choice._choice.Choice",

"text": "Select the type of image to save",

"value": "Image"

},

{

"name": "cellprofiler_core.setting.subscriber.image_subscriber._image_subscriber.ImageSubscriber",

"text": "Select the image to save",

"value": "OrigOverlay"

},

{

"name": "cellprofiler_core.setting.choice._choice.Choice",

"text": "Select method for constructing file names",

"value": "From image filename"

},

{

"name": "cellprofiler_core.setting.subscriber.image_subscriber._file_image_subscriber.FileImageSubscriber",

"text": "Select image name for file prefix",

"value": "Gb3"

},

{

"name": "cellprofiler_core.setting.text._text.Text",

"text": "Enter single file name",

"value": "OrigBlue"

},

{

"name": "cellprofiler_core.setting.text.number.integer._integer.Integer",

"text": "Number of digits",

"value": "4"

},

{

"name": "cellprofiler_core.setting._binary.Binary",

"text": "Append a suffix to the image file name?",

"value": "Yes"

},

{

"name": "cellprofiler_core.setting.text._text.Text",

"text": "Text to append to the image name",

"value": "object_overlay"

},

{

"name": "cellprofiler_core.setting.choice._choice.Choice",

"text": "Saved file format",

"value": "tiff"

},

{

"name": "cellprofiler.modules.saveimages.SaveImagesDirectoryPath",

"text": "Output file location",

"value": "Default Input Folder sub-folder|Desktop"

},

{

"name": "cellprofiler_core.setting.choice._choice.Choice",

"text": "Image bit depth",

"value": "16-bit integer"

},

{

"name": "cellprofiler_core.setting._binary.Binary",

"text": "Overwrite existing files without warning?",

"value": "No"

},

{

"name": "cellprofiler_core.setting.choice._choice.Choice",

"text": "When to save",

"value": "Every cycle"

},

{

"name": "cellprofiler_core.setting._binary.Binary",

"text": "Record the file and path information to the saved image?",

"value": "No"

},

{

"name": "cellprofiler_core.setting._binary.Binary",

"text": "Create subfolders in the output folder?",

"value": "No"

},

{

"name": "cellprofiler_core.setting.text._directory.Directory",

"text": "Base image folder",

"value": "Elsewhere...|"

},

{

"name": "cellprofiler_core.setting.choice._choice.Choice",

"text": "How to save the series",

"value": "T (Time)"

}

]

}

],

"version": "v6"

}
