# Supplementary material for: Automated quantification of skin Gb3 load and white matter lesion assessment in Fabry disease
Source: Orphanet J Rare Dis. 2026 Jul 7;21:245. doi: 10.1186/s13023-026-04490-4 (PMC13355356; doi:10.1186/s13023-026-04490-4)
Supplement: Supplementary file 5 — Supplementary Material 5 [file 13023_2026_4490_MOESM5_ESM.docx]

**Supplementary Table 1: *GLA* sequence variants and group allocation of study participants.**

| **Patients** | **Sequence variants** | **Group allocation** |
| --- | --- | --- |
| FD_1 | c.1029_1030 del TC fs*30 | G1 |
| FD_2 | c.155G>C // p.C52S | G1 |
| FD_3 | c.404C>T // p.A135V | G1 |
| FD_4 | c.927del // p.L310Sfs*8 | G1 |
| FD_5 | c.993_994 ins A fs*339 | G1 |
| FD_6 | c.806 T>G // p.V269G  c.937G>T // p.D313Y | G1 |
| FD_7 | c.188G>A // p.C63Y  c.1000-22C>T | G1 |
| FD_8 | c.162del T | G1 |
| FD_9 | c.756 or 757 del A fs*268 | G1 |
| FD_10 | c.408T>A // p.D136E | G1 |
| FD_11 | c.1208 del AAG | G1 |
| FD_12 | c.1208 del AAG | G1 |
| FD_13 | c.644A>G // p.N215S | G1 |
| FD_14 | deletion exon 2 | G1 |
| FD_15 | c.515G>A // p.C172Y | G1 |
| FD_16 | c.1021G>A // p.E341K | G1 |
| FD_17 | c.404C>T // p.A135V | G1 |
| FD_18 | c.644A>G // p.N215S | G1 |
| FD_19 | c.395 G>A // p.G132E | G1 |
| FD_20 | c.863delC // p.A288Vfs*29 | G1 |
| FD_21 | c.934C>T // p.Q312X | G1 |
| FD_22 | c.611G>A // p.W204X | G1 |
| FD_23 | c.679C>T // p.R227X | G1 |
| FD_24 | c.1072_1074del // p.E358del | G1 |
| FD_25 | c.708G>C // p.W236C | G1 |
| FD_26 | c.1196G>A // p.W399X | G1 |
| FD_27 | c.644A>G // p.N215S | G1 |
| FD_28 | c.994dup // p.R332Kfs*7 | G1 |
| FD_29 | c.369+1G>A | G1 |
| FD_30 | c.386T>C // p.L129P | G1 |
| FD_31 | c.993_994 ins A fs*338 | G1 |
| FD_32 | c.1046G>A // p.W349X | G1 |
| FD_33 | c.679C>T // p.R227X | G1 |
| FD_34 | c.386T>C // p.L129P | G1 |
| FD_35 | c.1069C>T // p.Q357X | G1 |
| FD_36 | c.1069C>T // p.Q357X | G1 |
| FD_37 | c.1000-1G>A | G1 |
| FD_38 | c.937G>T // p.D313Y | G1 |
| FD_39 | c.644 A>G // p.N215S | G1 |
| FD_40 | c.644 A>G // p.N215S | G1 |
| FD_41 | c.644 A>G // p.N215S | G1 |
| FD_42 | c.644A>G // p.N215S | G1 |
| FD_43 | c.644A>G // p.N215S | G1 |
| FD_44 | c.644A>G // p.N215S | G1 |
| FD_45 | c.644A>G // p.N215S | G1 |
| FD_46 | c.937G>T // p.D313Y | G2 |
| FD_47 | c.376A>G // p.S126G | G2 |
| FD_48 | c.427 G>A // p.A143T | G2 |
| FD_49 | c.427G>A // p.A143T | G2 |
| FD_50 | c.937G>T // p.D313Y | G2 |
| FD_51 | c.486G>T // p.W162C | G3 |
| FD_52 | c.1184G>C // p.G395A | G3 |
| FD_53 | c.1184G>C // p.G395A | G3 |
| FD_54 | c.720G>C // p.K240N | G3 |
| FD_55 | c.973G>A  // p.G325S | G3 |
| FD_56 | c.508G>A //  p.D170N | G3 |
| FD_57 | c.784T>C // p.W262R | G3 |
| FD_58 | c.1025G>T // p.R342L | G3 |
| FD_59 | c.1184G>C // p.G395A | G3 |
| FD_60 | c.508G>A //  p.D170N | G3 |
| FD_61 | c.515G>A // p.C172Y | G1 |
| FD_62 | c.994dup // p.R332Kfs*7 | G1 |
| FD_63 | c.644A>G // p.N215S | G1 |
| FD_64 | c.994dup // p.R332Kfs*7 | G1 |
| FD_65 | c.155G>C // p.C52S | G1 |
| FD_66 | c.416A>G // p.N139S  c.708G>C //  p.W236C | G1 |
| FD_67 | c.1046G>A // p.W349X | G1 |
| FD_68 | c.1046G>A // p.W349X | G1 |
| FD_69 | c.838 C>T // p.Q280X | G1 |
| FD_70 | c.973G>A // p.G325S | G1 |
| FD_71 | c.993_994 ins A fs*338 | G1 |
| FD_72 | c.806T>G // p.V269G  c.937G>T // p.D313Y | G1 |
| FD_73 | c.188G>A // p.C63Y  c.1000-22C>T | G1 |
| FD_74 | deletion exon 2 | G1 |
| FD_75 | c.334C>T // p.R112C | G1 |
| FD_76 | c.408T>A // p.D136E | G1 |
| FD_77 | c.1000-1G>A | G1 |
| FD_78 | c.756 or 757 del A, fs*268 | G1 |
| FD_79 | c.1223del // p.N408Ifs*10 | G1 |
| FD_80 | c.560T>G // p.M187R | G1 |
| FD_81 | c.973G>A // p.G325S | G1 |
| FD_82 | c.1072_1074del // p.E358del | G1 |
| FD_83 | c.718_719del // p.K240Efs*9 | G1 |
| FD_84 | c.350T>G // p.I117S | G1 |
| FD_85 | c.404C>T // p.A135V | G1 |
| FD_86 | c.369+1G>A | G1 |
| FD_87 | c.404C>T // p.A135V | G1 |
| FD_88 | c.1223del // p.N408Ifs*10 | G1 |
| FD_89 | c.1196G>A // p.W399X | G1 |
| FD_90 | c.363del // p.N122Ifs*8 | G1 |
| FD_91 | c.927del // p.L310Sfs*7 | G1 |
| FD_92 | c.644A>G // p.N215S | G1 |
| FD_93 | c.644A>G // p.N215S | G1 |
| FD_94 | c.386T>C // p.L129P | G1 |
| FD_95 | c.1196G>A // p.W399X | G1 |
| FD_96 | c.757del // p.I253Lfs*16 | G1 |
| FD_97 | c.757del // p.I253Lfs*16 | G1 |
| FD_98 | c.1069C>T // p.Q357X | G1 |
| FD_99 | c.1208 del AAG | G1 |
| FD_100 | c.648T>A // p.Y216X | G1 |
| FD_101 | c.648T>A // p.Y216X | G1 |
| FD_102 | c.1000-1G>A | G1 |
| FD_103 | c.515G>A // p.C172Y | G1 |
| FD_104 | c.874G>C // p.A292P | G1 |
| FD_105 | c.137A>G // p.H46R | G1 |
| FD_106 | c.1196G>A // p.W399X | G1 |
| FD_107 | c.404C>T // p.A135V | G1 |
| FD_108 | c.404C>T // p.A135V | G1 |
| FD_109 | c.644A>G // p.N215S | G1 |
| FD_110 | c.644A>G // p.N215S | G1 |
| FD_111 | c.644A>G // p.N215S | G1 |
| FD_112 | c.644A>G // p.N215S | G1 |
| FD_113 | c.644A>G // p.N215S | G1 |
| FD_114 | c.644 A>G // p.N215S | G1 |
| FD_115 | c.644A>G // p.N215S | G1 |
| FD_116 | c.937G>T // p.D313Y | G2 |
| FD_117 | c.937G>T // p.D313Y | G2 |
| FD_118 | c.937G>T // p.D313Y | G2 |
| FD_119 | c.937G>T // p.D313Y | G2 |
| FD_120 | c.-110-15T>G | G2 |
| FD_121 | c.427G>A // p.A143T | G2 |
| FD_122 | c.1196G>C // p.W399S  c.370-10C>T  c.370-81_370-77del  c.640-16A>G  c.1000-22C>T | G2 |
| FD_123 | c.427G>A // p.A143T | G2 |
| FD_124 | c.376A>G // p.S126G | G2 |
| FD_125 | c.352C>T // p.R118C | G2 |
| FD_126 | c.937G>T // p.D313Y | G2 |
| FD_127 | c.937G>T // p.D313Y | G2 |
| FD_128 | c.427G>A // p.A143T | G2 |
| FD_129 | c.937G>T // p.D313Y | G2 |
| FD_130 | c.427G>A // p.A143T | G2 |
| FD_131 | c.937G>T // p.D313Y | G2 |
| FD_132 | c.640-16A>G  c.1000-22C>T | G2 |
| FD_133 | c.427G>A // p.A143T | G2 |
| FD_134 | c.937G>T // p.D313Y | G2 |
| FD_135 | c.35_58del // p.C12_A20delinsS | G3 |
| FD_136 | c.1250T>C // p.L417P | G3 |
| FD_137 | c.354_368del // p.Q119_Y123del | G3 |
| FD_138 | c.802-3_802-2del | G3 |
| FD_139 | c.1025G>T // p.R342L | G3 |
| FD_140 | c.1184G>C // p.G395A | G3 |
| FD_141 | c.612G>T // p.W204C  c.370-10C>T  c.1000-22C>T | G3 |
| FD_142 | c.1025G>T // p.R342L | G3 |
| FD_143 | c.860G>C // p.W287S | G3 |
| FD_144 | c.1250T>C // p.L417P | G3 |
| FD_145 | c.408T>A // p.D136E | G3 |
| FD_146 | c.1025G>T // p.R342L | G3 |
| FD_147 | c.1184G>C // p.G395A | G3 |
| FD_148 | c.515G>A // p.C172Y | G3 |
| FD_149 | IVS2-81.-77 + IVS0-10C>T, IVS4-16A>G, IVS6-22 C>T | G3 |

**Abbreviations:** FD: Fabry disease, *GLA*: alpha-galactosidase A gene.

G1: pathogenic *GLA* variants, G2: non-pathogenic *GLA* variants, G3: variants of unknown significance.
